# Inertial motion of a circular cylinder approaching obliquely an ice cover

**DOI:** 10.1038/s41598-025-93435-1

**Published:** 2025-03-19

**Authors:** Hang Xiong, Baoyu Ni, Yuriy Semenov, Alexander Korobkin

**Affiliations:** 1 College of Shipbuilding Engineering, Harbin Engineering University, Harbin, 150001 People’s Republic of China; 2 School of Engineering, Mathematics and Physics, University of East Anglia, Norwich Research Park, Norwich, NR4 7TJ UK

**Keywords:** Inertial motion, Ice sheet, Ice breaking, Physical oceanography, Civil engineering

## Abstract

Two-dimensional unsteady problem of an inertial motion of a circular cylinder approaching obliquely an ice cover and the response of ice to this motion are investigated. The liquid under the ice is inviscid, incompressible and of infinite depth. The ice sheet floating on water surface is modelled as a thin elastic plate of constant thickness and of infinite extent. The movement of the cylinder is governed by its inertia, gravity, hydrodynamic force, deflection of the ice cover and the initial conditions. The general coupled problem is approximately decoupled for relatively small speeds of the body. Within the decoupled approach, the cylinder motion, the generated flow, and the hydrodynamic pressure in the fluid are determined by conformal mapping method without account for the ice deflection. The obtained hydrodynamic loads are applied to the equation of elastic ice sheet, and the ice deflection, speed of deflection and strains in the ice are evaluated by Fourier transform method. The oblique inertial motion of a circular cylinder under the rigid plate is described analytically. A critical Froude number for a heavy cylinder, which is only dependent on the initial submergence depth, is introduced and used in classification of the body motions. The present study is focused on the motions of circular cylinders without their impacts with the plate. It is shown that the ice can be damaged even before the cylinder arrives at the position closest to the plate. For a given radius of the cylinder and its initial kinetic energy, the conditions of the motion including the angle of attack and the dimensionless submergence depth which lead to ice breaking are predicted.

## Introduction

**Fig. 1:**
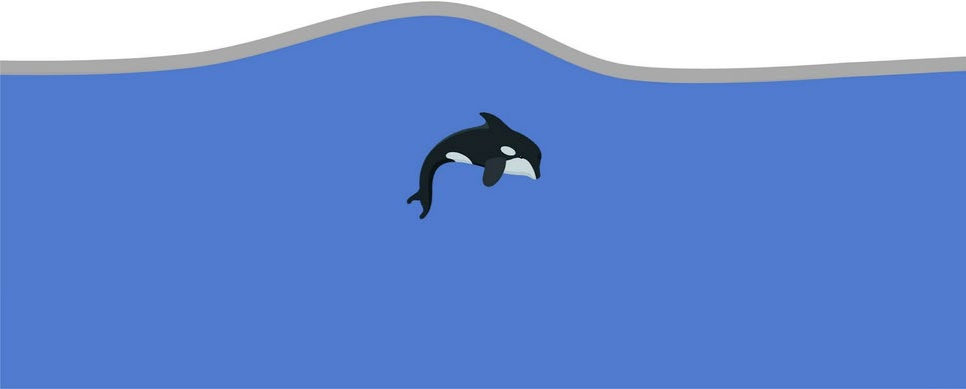
Ice deformation caused by a killer whale.

Events have been witnessed in nature that in order to hunt for prey such as seals perched on top of the ice cover, killer whales moved underneath the ice in an undulating motion, which created waves that broke up the ice, ultimately causing the prey to fall into the water^1^, see Fig. 1. This phenomenon corresponds in scientific research to the familiar idea that ice cover can be weakened or even broken by flexural-gravity waves generated by a body moving either under or on the ice cover^2,3^. This was first shown theoretically by Kheisin (1967)^4^ and later experimentally by Kozin and Onishchuk (1994)^5^.

In theoretical studies, point sources are often used to model objects moving horizontally under the ice cover. The effects of both the distance of the object from the ice and the speed of the object on the deflection of the ice sheet were investigated for constant low speeds of the object by Bukatov and Zharkov (1995), Kozin and Pogorelova (2008), Pogorelova and Kozin (2014)^6–8^, and for variable speed by Pogorelova and Kozin (2010)^9^ and Pogorelova (2011)^10^. Bukatov and Zharkov (1995)^6^ studied flexural-gravity waves on the surface of continuously stratified liquid of a finite depth caused by a point source of constant intensity moving under the ice at a constant speed. The ice deflection was stationary in the coordinate system moving together with the source. The pattern of the ice deflection above the source was studied depending on the ice thickness, the speed of the source and its submergence depth. There are several critical speeds for a continuously stratified liquid, which makes this problem complicated. Kozin and Pogorelova (2008)^7^ considered the problem of a point source moving at constant speed in water of infinite depth covered by a floating elastic plate. They found that the inertia of the ice sheet is negligible compared with the elastic forces for low speeds of the source but cannot be neglected for supercritical speeds, where the dynamic effects are well pronounced. They suggested to weaken continuous ice by flexural-gravity waves before submarine surfacing. Pogorelova and Kozin (2010)^9^ further investigated the effects of some parameters on the response of ice sheets to a point source moving at a variable speed in a liquid of finite depth under a floating elastic plate. It was shown that the ice deflection decreases when the ice thickness, Young’s modulus and/or the submergence depth increase. Note that linear models of hydroelasticity without account for viscous properties of the ice and nonlinear effects predict unbounded ice deflection for an object moving at a critical speed, which is the speed at which the group and phase velocities of the flexural-gravity waves are equal, see Squire et al. (1996)^11^. The flexural-gravity waves are not generated by a body moving at subcritical speed. A point source represents a body elongated to infinity in the direction opposite to the direction of the source motion. The shape of such a body depends on all characteristics of the problem. This shape was not investigated but it is believed that it can model the front part of a submarine. Note that Kheisin (1967)^4^ investigated the two-dimensional problem of a point vortex moving under the water surface covered by broken ice. The effect of the broken ice on waves generated by the point vortex was found to be minor.

A slender body moving at a constant speed in unbounded liquid can be modeled by a source-sink system. This idea was used by Pogorelova et al. (2012)^12^ to calculate the deflections of an elastic floating plate caused by a submarine moving at variable speed under the plate. The obtained numerical results were compared with results of experiments with a polymer floating plate and a model of a submarine. “Good qualitative agreement with the results of the model experiment” was reported. More accurate representations of the actual shape of a body moving under ice cover can be achieved by using several sinks and sources of different intensities, see Pogorelova et al. (2023)^13^, where calculations with one source and ten sinks were performed for a particular shape of the body and were compared successfully in terms of the wave resistance with the experimental results by Dawson (2014)^14^. Sturova (2012)^15^ solved the problem of a sphere moving horizontally at constant speed under a compressed elastic plate by the method of multipole expansions. Sturova (2013)^16^ derived a three-dimensional velocity potential of the flow caused by an arbitrary motion of a point source of time-dependent strength under elastic ice cover. Using the obtained velocity potential and the method of multipole expansions, the flexural-gravity waves generated by a rigid sphere moving horizontally at a constant speed were studied.

A moving circular cylinder and a sphere in unbounded liquid can be modeled by a two-dimensional and three-dimensional dipole of a certain strength correspondingly. Dipoles can be used to approximately describe flow fields generated by a circular cylinder or sphere moving under the ice cover. This approximation is accurate enough if a body is far from the ice. Two-dimensional unsteady problem of ice deflection caused by a dipole moving beneath ice plate in water of infinite depth was studied by Savin and Savin (2012)^17^ and Il’ichev et al. (2012)^18^. Large-time asymptotic behaviour of the solution in the moving coordinate system was obtained. The corresponding three-dimensional problem was studied by Savin and Savin (2015)^19^. Shishmarev et al. (2017, 2019)^20,21^ studied a dipole moving in a channel of finite width and depth covered by ice sheet of constant thickness. The ice sheet was modeled as a thin viscoelastic plate clamped to the vertical walls of the channel. The dipole moved along the channel at a constant speed. Several models of ice response were considered. It was shown that the problem can be approximately decoupled for dipoles of small strength, which is for small bodies. Within the decoupled approach, the problem of a dipole moving in the channel with rigid cover was solved. Then the obtained pressure distribution was applied to the elastic plate floating on the surface of the liquid. The resulting problem was solved by the normal mode method and the Fourier transform along the channel. Distributions of the deflections and strains in the ice were investigated for different parameters of the problem. Stepanyants and Sturova (2021)^22^ considered a two-dimensional dipole moving along an arbitrary path in a liquid beneath a compressed elastic plate.

Korobkin et al. (2012)^23^ and Kostikov et al. (2018)^24^ investigated nonlinear unsteady problem of an elliptic cylinder moving under an ice cover. Initial solution of this problem for small times and large distances between the ice and the cylinder was obtained analytically. Deflection of the ice plate was analysed and compared to the elevation of the water surface without the ice cover.

Relevant problems with stationary bodies under ice cover subject to incident flexural-gravity waves were considered by Das and Mandal (2006, 2008)^25,26^ for a circular cylinder and a sphere correspondingly, and subject to an underwater uniform current by Li et al. (2019)^27^ and Yang et al. (2021)^28^ in two- and three-dimensional cases correspondingly. The nonlinear problem of a stationary two-dimensional body in a uniform current under a floating ice sheet was solved by Semenov (2021)^29^. Ni et al. (2024)^30^ further investigated this problem for an obstacle on the bottom.

Three-dimensional linear problems of a rigid elongated body moving under ice cover were solved numerically by Kozin et al. (2010)^31^ and Zemlyak et al. (2019)^32^. A finite-element method was used to describe the dynamics of the ice plate and the boundary-element method was employed to solve the hydrodynamic part of the problem. Ice cover was modeled as a thin viscoelastic plate of constant thickness. Numerical results were compared with the results of laboratory experiments performed by the authors. It was concluded that the developed mathematical model of a body moving under an ice cover well describes the results of the measurements. The effects of the body shape, submergence depth and properties of the ice cover on the ice deflection were studied by Zemlyak et al. (2013, 2018, 2019, 2022)^32–35^ and Pogorelova et al. (2019)^36,37^.

Ni et al. (2023)^38^ performed experiments with a buoyant sphere free-rising towards the ice cover from a certain depth. This study was focused on the collision between the sphere and the ice, and the following cracking the ice cover.

We may conclude that the main part of research on ice cover deflection caused by a body moving under the ice was done for horizontal motion of the body at constant speed. Small periodic perturbations of the body speed were studied by Stepanyants and Sturova (2021)^39^. Large-amplitude oscillations of a submerged circular cylinder under an ice cover were investigated by Li et al. (2017)^40^ assuming small deflections of the ice. All problems were studied as coupled problems with the ice deflection, the hydrodynamic loads and the flow characteristics being determined at the same time. The parameters of the problem, which allow to treat the problem as decoupled, were not well investigated yet. It is not clear also for which conditions the ice dynamics caused by a moving underwater body can be treated as linear.

It is known that ice is a brittle material at low temperature. This imply that the strains in an ice cover, which behaves elastically, should be very small before the ice breaking. Therefore, the slope of the ice plate should be also small, which leads to the conclusion that, if the ice breaks, it occurs in the linear elastic region. This conlcusion was supported by Ni et al (2024)^30^, where nonlinear flexural-gravity waves caused by an obstacle in an uniform current were studied, see figure 14 there. The obtained results confirmed that the strains in the ice cover do not exceed the yield strain value for the ice only for very small obstacles, where the linear theory of flexural-gravity waves can be applied.

Another field of research, which is relevant to the present study and has its own important applications, deals with bodies moving close to solid stationary walls. Miloh (1977)^41^ solved the problem of two spheres moving arbitrary in an unbounded inviscid fluid and a sphere moving between two rigid parallel plates. The forces between the spheres were obtained using series of spherical harmonics with the coefficients being the solutions of an infinite system of linear equations. Eames et al. (1996)^42^ studied the flow caused by a rigid sphere moving away from a flat wall in an unbounded inviscid fluid. This study was concerned with displacements of liquid particles around the moving sphere. The velocity potential of the flow was sought as a series with respect to the associated Legendre polynomials. The coefficients of the series satisfied a recurrence relation derived by Kok (1993)^43^ and Li et al. (1993)^44^. It was noted that the resulting series converges quickly only if the distance between the wall and the sphere is greater than the radius of the sphere. Motions of spheroidal particles in a shear viscous flow close to rigid wall were intensively studied numerically, see Gavze (1998)^45^, for example.

Two-dimensional problem of unsteady motion of two circular cylinders in an unbounded inviscid fluid was studied by Wang (2004)^46^ using a conformal mapping of the flow region onto a circular ring. The original problem was reduced to a Neumann problem for Laplace’s equation in a circular ring. The unsteady forces acting between the cylinders were evaluated. It was shown that the forces become unbounded when the cylinders are approaching each other. If the radius of one of the cylinders tends to infinity in the solution, one arrives at the problem for a circular cylinder moving near a flat wall. The method of Wang (2004)^46^ was generalized by Tchieu et al. (2010)^47^ to motions of two arbitrary two-dimensional bodies in an unbounded fluid if a conformal mapping of the flow region onto a circular ring is known at each time instant. Circulations around the bodies were zero in this analysis. Motion of a circular cylinder near a flat wall with a circulation was studied by Petrov and Maklakov (2022)^48^.

In the present paper, we investigate the problem of a circular cylinder moving inertially towards a floating elastic plate from below. The problem is formulated in dimensionless coordinates and the decoupled approach is discussed in section “Formulation of the problem”. The conditions, under which the cylinder motion can be approximately determined without account for the elastic plate deflection, are obtained. In section “Method”, the leading order solution of the pressure field, the cylinder motion, the ice response including deflection, speed of deflection, and strain of the ice plate are obtained. The inertial oblique motion of the cylinder and the ice response caused by inertial motion of a submerged cylinder are discussed and analyzed with the numerical results in section “Results”. Conclusions are drawn in section “Conclusion”.

**Fig. 2:**
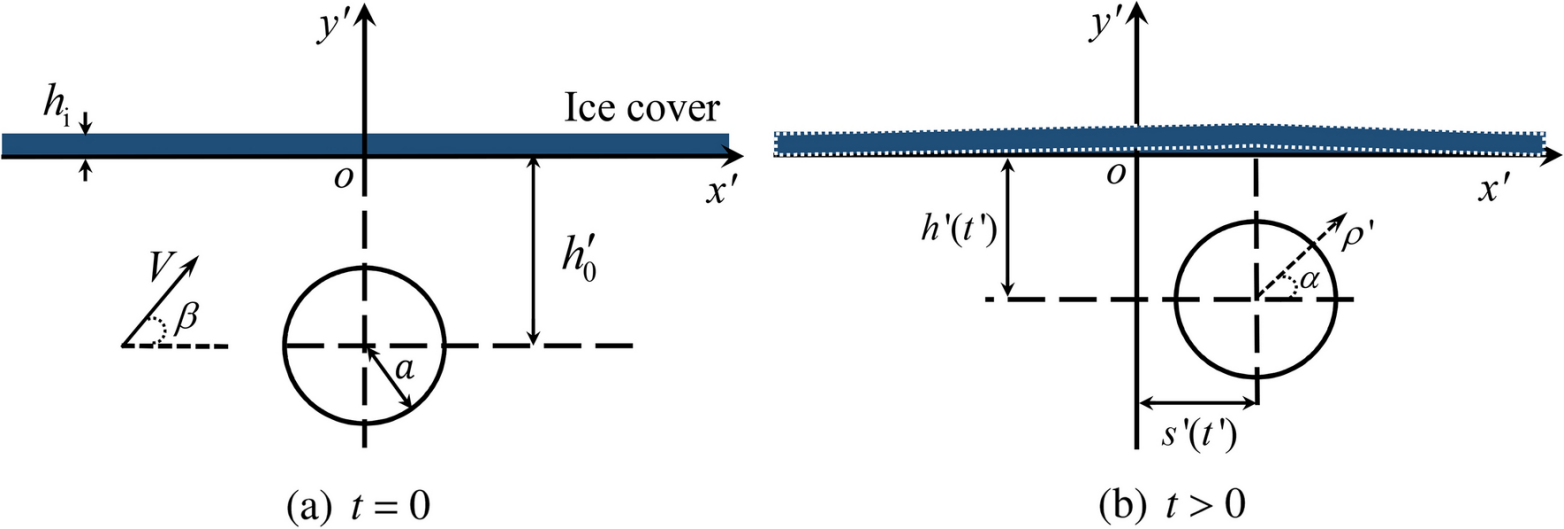
Sketch of the problem and notation.

## Formulation of the problem

Motion of a circular cylinder beneath ice cover and the strains generated in the ice due to this motion are studied. The ice sheet floating on the water surface is modelled as a thin elastic plate of constant thickness $h_i$ and of infinite extent. The liquid flow under the ice and the deflection of the ice cover are described in the Cartesian coordinate system $Ox'y'$, see Fig. 2. A prime stands for dimensional variables. The line $y'=0$ corresponds to the initial position of the ice/water interface at $t'=0$. The liquid under the ice is inviscid, incompressible and of infinite depth. A cylinder of radius *a* is placed at $t'=0$ under the ice with the centre of the cylinder being at $x'=0$, $y'=-h'_0$, see Fig. 2a. The cylinder is rigid. The cylinder starts moving towards the ice cover at $t'=0$ with an initial speed *V*. The cylinder is launched at an angle $\beta$ to the horizontal with the initial horizontal and vertical velocity components being $V\cos \beta$ and $V\sin \beta$ correspondingly, see Fig. 2a. The motion of the cylinder is governed by its inertia, gravity, hydrodynamic force, deflection of the ice cover and the initial velocity. The liquid flow caused by the cylinder motion is assumed two-dimensional and potential. The corresponding velocity potential $\varphi '(x',y',t')$ satisfies the Laplace equation in the flow region $\Omega '(t')$, the boundary conditions on the surface of the moving cylinder and on the ice/liquid interface, and decays in the far field as ${x'^2}+{y'^2} \rightarrow \infty$. The hydrodynamic pressure $p'(x',y',t')$ in $\Omega '(t')$ is given by the nonlinear and unsteady Bernoulli equation. To formulate the boundary condition on the surface of the cylinder, it is convenient to introduce the moving polar coordinates, $\rho '$, $\alpha$, where $x'=s'(t')+\rho ' \text{ cos }\alpha$ and $y'=-h'(t')+\rho '\text{ sin }\alpha$, $\rho ' \ge a$ and $0<\alpha <2\pi$. Here $s'(t')$ is the horizontal displacement of the cylinder, and $h'(t')$ is the distance of the cylinder centre from the *x*-axis. Initially $s'(0)=0$, $h'(0)=h'_0$, $\text{d}s'/\text{d }t'(0)=V\cos \beta$ and $\text{d}h'/\text{d }t'(0)=-V\sin \beta$, see Fig. 2b.

In general, this problem is coupled because the ice deflection depends on the hydrodynamic loads and the motion of the cylinder, which are, in turn, depend on the ice deflection through the kinematic condition on the ice/water interface. We shall find conditions, under which the original problem can be approximately decoupled. Within the decoupled formulation, the hydrodynamic loads and the motion of the cylinder are determined for rigid ice sheet. Next, the obtained hydrodynamic loads are applied to the equation of elastic ice sheet, and the ice deflection and strains in the ice are evaluated.

The problem is formulated in dimensionless variables, where the radius of the cylinder *a* is taken as the length scale, *a*/*V* as the time scale, the product *Va* as the scale of the velocity potential and $\rho _w V^2$ as the scale of the hydrodynamic pressure, where $\rho _w$ is the water density. The shape of the ice/water interface is described by the equation $y'=w'(x',t')$, where $w'(x',t')$ is the ice deflection. The scale of the ice deflection $w_{sc}$ is obtained by requiring the balance between the order of the hydrodynamic pressure acting on the ice sheet, $\rho _w V^2$, and the order of the bending term in the elastic plate equation, $D {\partial ^4 w'}/{\partial x'^4}=O(Dw_{sc}/a^4)$, where $D = {{E{h_i}^3} / {\left[ {12(1 - {\nu ^2})} \right] }}$ is the rigidity of the ice plate, *E* is the Young modulus and $\nu$ is the Poisson ratio of the ice. The balance of these two terms gives $w_{sc}=\rho _w V^2 a^4 /D$. The dimensionless variables are denoted by the same symbols but without primes.

We assume that the conditions of the motions are such that the deflection scale, $w_{sc}$, is much smaller than the length scale of the problem, *a*. Therefore, $\varepsilon =w_{sc}/a$ is a small parameter of the problem, and $\varepsilon \ll 1$. This condition provides,

1 $$\begin{aligned} V \ll {c_i} \left( \frac{\rho _i}{\rho _w} \right) ^{1/2} \left( \frac{h_i}{a} \right) ^{3/2} \frac{1}{[12(1-\nu ^2)]^{1/2}}, \end{aligned}$$

where $c_i=\sqrt{E/\rho _i}$ is the speed of longitudinal waves in the ice and $\rho _i$ is the ice density. It is seen that the condition (1) is satisfied only for relatively small speeds of the cylinder.

In the present study, the following reference values of the parameters are used,

2 $$\begin{aligned} \begin{array}{l} \rho _i=917\, \mathrm{kg/m}^3,\,\, E=4.2\times 10^9 \,\mathrm{N/m}^2,\,\, \nu =0.3, \\ h_i=0.2\,\textrm{m},\,\, a=0.5\,\textrm{m},\,\, \rho _w=1000 \, \mathrm{kg/m}^3, \end{array} \end{aligned}$$

which gives $c_i=2140 \,\mathrm{m/s}$. The inequality (1) yields $V\ll 150 \,\mathrm{m/s}$. We conclude that for a cylinder of diameter $1\,\textrm{m}$ and ice thickness of $20\, \textrm{cm}$, the ice deflection is much smaller than the cylinder radius if the initial speed of the cylinder is much smaller than $150 \,\mathrm{m/s}$. The estimate (1) is from above because the maximum hydrodynamic loads acting on the ice sheet are expected when the cylinder is at a short distance from the ice, but then the speed of the cylinder would be significantly reduced due to the water resistance. It should be noted that the reference speed of $150 \,\mathrm{m/s}$ is 1.5 times higher than the speed of the fastest existing underwater torpedo (200 knots). The speed of the cylinder in our study is additionally limited by the employed hydrodynamic model, where the liquid is assumed incompressible and not cavitating.

In the dimensionless variables, the velocity potential $\varphi (x,y,t)$ satisfies the Laplace equation,

3 $$\begin{aligned} \nabla ^2\varphi =0, \end{aligned}$$

in the flow region, $\Omega (t)=\{ x, y \,| \,y<\varepsilon w(x, t),\,\rho >1\}$, the kinematic boundary condition on the ice/water interface,

4 $$\begin{aligned} \varphi _y=\varepsilon (w_x \varphi _x+ w_t) \qquad (y=\varepsilon w(x,t)), \end{aligned}$$

the condition on the surface of the moving cylinder,

5 $$\begin{aligned} \varphi _\rho ={\dot{s}}\cos \alpha - {\dot{h}}\sin \alpha \qquad (\rho =1, 0\le \alpha \le 2\pi ), \end{aligned}$$

and decays at infinity,

6 $$\begin{aligned} \varphi \rightarrow 0 \qquad (x^2+y^2 \rightarrow \infty ). \end{aligned}$$

Here an overdot stands for time derivative, ${\dot{s}}=\text{d}s/\text{d }t$, ${\dot{h}}=\text{d}h/\text{d }t$. The dimensionless hydrodynamic pressure is given by the nonlinear Bernoulli equation,

7 $$\begin{aligned} p(x,y,t) = - \frac{\partial \varphi }{\partial t} -\frac{1}{2}{{\left| {\nabla \varphi } \right| }^2} - \frac{1}{{\textrm{Fr}}^2}y. \end{aligned}$$

where ${{\textrm{Fr}}}=V/\sqrt{ga}$ is the Froude number and *g* is the gravitational acceleration, $g=9.81 \,\mathrm{m/s}^2$.

The motion of the cylinder is governed by Newton’s second law,

8 $$\begin{aligned} M \frac{\text{ d}^2 s}{\text{d}t^2}=- \frac{1}{\pi }\int \limits _0^{2\pi } {p(1,\alpha ,t)\cos \alpha \text{d}\alpha }, \end{aligned}$$

9 $$\begin{aligned} M \frac{\text{ d}^2 h}{\text{d}t^2}=\frac{1}{\pi }\int \limits _0^{2\pi } {p(1,\alpha ,t)\sin \alpha \text{d}\alpha }+M{{\textrm{Fr}}^{-2}}, \end{aligned}$$

where $s'(t')=as(t)$, $h'(t')=ah(t)$, *M* is the ratio of the mass of the cylinder $M'$ to the mass of the water in the volume of the cylinder, $\pi a^2 \rho _w$, per unit width, $M=M'/(\pi a^2 \rho _w)$. The pressure $p(1,\alpha ,t)$ in (8) and (9) is written in the local moving polar coordinates $\rho$, $\alpha$, where the surface of the cylinder is at $\rho =1$. Equations (8) and (9) should be integrated in time subject to the initial conditions,

10 $$\begin{aligned} s(0)=0, \,\, \frac{\text{d}s}{ \text{d}t}(0)=\cos \beta ,\,\, h(0)=h_0,\,\, \frac{\text{d}h}{\text{d}t}(0)=-\sin \beta ,\,\, h_0=h_0'/a. \end{aligned}$$

The dimensionless deflection of ice sheet is described by the Kirchhoff equation of thin elastic plate,

11 $$\begin{aligned} {\varepsilon \chi }\frac{\partial ^2 w}{\partial t^2} + \frac{\partial ^4 w}{\partial x^4}= p(x, \varepsilon w(x,t), t) \qquad ( |x|<\infty ,t>0), \end{aligned}$$

with the initial,

12 $$\begin{aligned} w(x,0)=\partial w/ \partial t(x,0)= 0, \end{aligned}$$

and the far field,

13 $$\begin{aligned} w(x,t) \rightarrow 0 \qquad (x \rightarrow \pm \infty ), \end{aligned}$$

conditions, where $\chi =\rho _i h_i/(\rho _w a)$. The elastic strains take their maximum values on the upper and lower surface of the ice sheet, where

14 $$\begin{aligned} {\epsilon }(x,t)=\mp {\epsilon _{sc}} \frac{\partial ^2 w}{\partial x^2}, \end{aligned}$$

${{\epsilon _{sc}}}=\varepsilon h_i/{(2a)}$, minus in (14) is for the upper surface of the ice sheet and plus for the lower ice surface. The strain is positive, where the surface is in tension, and negative, where the ice surface is compressed. The ice sheet is assumed to be broken then and there, when and where the strain is positive and exceeds the so-called yield strain of the ice ${\epsilon _{Y}}$. Here we take ${\epsilon _{Y}}=8\times 10^{-5}$, see Brocklehurst et al. (2010)^49^ and discussions there.

The problem (3)–(13) is coupled. The flow under the ice, the hydrodynamic pressure, the motion of the cylinder and the ice deflection should be determined at the same time. However, for small values of the parameter $\varepsilon$, the kinematic boundary condition (4) can be approximated in the leading order by the condition of the rigid wall, $\varphi _y=0$, where $y=0$. This approximation makes the original problem decoupled: the flow, cylinder motion, and the hydrodynamic pressure in $y<0$ are determined without account for the ice deflection, and next the ice deflection is determined in the leading order by solving problem (11)–(13), where the external pressure *p*(*x*, 0, *t*) has been obtained solving the problem of hydrodynamics with the rigid wall at $y=0$. Note that the parameter $\varepsilon$ is proportional to $V^2$ and is equal to $4 \times 10^{-3}$ for the conditions (2) and the initial speed of the cylinder $V=10\, \mathrm{m/s}$. The decoupled approach could be not applicable to the motion of the cylinder in a close proximity of the ice sheet, where the gap between the surface of the cylinder and the deformed ice sheet is comparable with the deflection of the ice.

The formulated problem is studied for $\varepsilon \rightarrow 0$ with ${{\textrm{Fr}}}=O(1)$, $\chi =O(1)$ and $M=O(1)$. All unknown functions, $\varphi (x,y,t)$, *p*(*x*, *y*, *t*), *w*(*x*, *t*), *s*(*t*) and *h*(*t*) are assumed of order *O*(1) and having non-zero limits as $\varepsilon \rightarrow 0$. We limit ourselves to the second-order approximation of the solution, where the terms of order $O(\varepsilon ^2)$ and higher are neglected, and the flow region is approximated by $\Omega _2(t)=\{ x, y \,| \,y<0,\,\rho >1\}$. Note that the region $\Omega _2(t)$ is still dependent of $\varepsilon$ through the relations between the Cartesian coordinates *x*, *y* and the local polar coordinates $\rho$, $\alpha$.

The derivative on the left hand side in the condition (4) is approximated as,

15 $$\begin{aligned} \begin{aligned} \varphi _y(x,\varepsilon w(x,t),t)&=\varphi _y(x,0,t)+\varphi _{yy}(x,0,t)\varepsilon w(x,t)+ O(\varepsilon ^2) \\&=\varphi _y(x,0,t)-\varepsilon \varphi _{xx}(x,0,t) w(x,t)+ O(\varepsilon ^2), \end{aligned} \end{aligned}$$

where the Laplace equation (3) has been used. Corresponding, (4) takes the form

16 $$\begin{aligned} \varphi _y=\varepsilon \left( (w \varphi _x)_x + w_t \right) + O(\varepsilon ^2) \qquad (y=0). \end{aligned}$$

By using expansions similar to (15) as $\varepsilon \rightarrow 0$, the right hand side of the elastic plate equation (11) can be approximated as

17 $$\begin{aligned} \begin{aligned} p(x,\varepsilon w(x,t),t)&=-\displaystyle \frac{\varepsilon }{\mathrm{{Fr}}^2} w(x,t) -\displaystyle \frac{\partial \varphi }{\partial t}(x,\varepsilon w(x,t),t)- \frac{1}{2}|\nabla \varphi |^2(x,\varepsilon w(x,t),t)\\&=-\displaystyle \frac{\varepsilon }{\textrm{Fr}^2} w(x,t)-\displaystyle \frac{\partial \varphi }{\partial t}(x,0,t)-\displaystyle \frac{1}{2} \varphi _x^2(x,0,t)+ O(\varepsilon ^2), \end{aligned} \end{aligned}$$

where we used that $\varphi _y(x,0,t)=O(\varepsilon )$, see (16). Equations (8), (9) of the cylinder motion are not simplified as $\varepsilon \rightarrow 0$.

To determine the second-order approximation of the solution as $\varepsilon \rightarrow 0$, we solve the original problem (3)–(13) in two steps. First, we solve the hydrodynamic part of the problem (3)–(7) for $\varepsilon =0$ together with the equations of the body motion (8), (9) and the initial conditions (10). The obtained leading-order velocity potential is substituted in (17) and the structural part of the problem, (11)–(13), is solved for the given time-dependent external load. In this way, we determine the leading-order deflection of the ice plate and the strains in it. Note that we keep the terms in (11) corresponding to the ice inertia and the hydrostatic pressure, both of them being of order $O(\varepsilon )$ as $\varepsilon \rightarrow 0$ even in the leading order problem. A reason for this is that Eqs. (11) and (17) provide the deflection which satisfies the far-field condition (13). The formal application of the asymptotic analysis, where the first term in (17) and the first term on the left-hand side of (11) are neglected at the leading order as $\varepsilon \rightarrow 0$, would require the leading order solution in the far field, $|x|\gg 1$, and matching it with the solution in the near field, $|x|=O(1)$. At the second step, we substitute the leading-order deflection and velocity potential in the right hand side of the kinematic boundary condition (16) and solve the hydrodynamic problem (3), (16), (5) and (6) together with the equations of the body motion (8), (9) and the initial conditions (10) again but now with a non-zero normal derivative $\varphi _y(x,0,t)$ on the upper boundary of the flow region. This gives us the velocity potential and the body motion with accuracy $O(\varepsilon ^2)$. Next, the updated velocity potential is substitute in (17) and the plate equation (11) is solved again with updated forcing and the original initial (12) and far-field (13) conditions.

In the present paper, we limit ourselves to the leading-order motion of the cylinder and the ice plate deflection. However, the algorithm described in the next section is general and can be used to determine the first-order correction of the solution as it is explained above.

## Method

In the leading-order as $\varepsilon \rightarrow 0$, one should solve the hydrodynamic problem (3)–(6), where $\varepsilon$ is set zero, determine the pressure field using the Bernoulli equation (7) and integrate the equations (8), (9) of the cylinder motion in time together with the initial conditions (10), and finally applied hydrodynamic pressure (17) to the elastic plate equation (11), and solve this equation subject to the initial (12) and far-field (13) conditions. Note that $\varepsilon$ is set to zero only in the hydrodynamic part of the original program. Correspondingly, the solution with accuracy $O(\varepsilon ^2)$ is given by the same procedure, as it is described for the leading-order solution, with the only difference that now the kinematic boundary condition on the ice/water interface has the form (16), where *w*(*x*, *t*) and $\varphi _x(x,0,t)$ in the right hand side are given by the leading-order solution and $\varepsilon \ne 0$. Only the leading-order solution is considered in this section.

### Velocity potential of the flow

Within the leading-order approximation as $\varepsilon \rightarrow 0$, the flow caused by the moving cylinder is described by the velocity potential $\varphi (x,y,t)$, which satisfies the Laplace equation,

18 $$\begin{aligned} \nabla ^{2}\varphi = 0 \qquad ((x,y) \in \Omega _2(t)), \end{aligned}$$

and the boundary conditions,

19 $$\begin{aligned} \varphi _y= 0 \qquad (y=0,|x|<\infty ), \end{aligned}$$

20 $$\begin{aligned} \varphi _\rho = {\dot{s}}(t) \cos \alpha - {\dot{h}}(t)\sin \alpha \qquad (\rho =1, 0\le \alpha \le 2\pi ), \end{aligned}$$

21 $$\begin{aligned} \varphi \rightarrow 0 \qquad (x^2+y^2 \rightarrow \infty ) \end{aligned}$$

where the dimensionless Cartesian *x*, *y*, and local polar coordinates $\rho$, $\alpha$ are related by $x=s(t)+\rho \cos \alpha$, $y=-h(t)+\rho \sin \alpha$. Note that $h(t)>1$ when the cylinder is well under the ice and $h(t)=1$ when the cylinder touches the ice.

The boundary-value problem (18)–(21) is solved using the conformal mapping,

22 $$\begin{aligned} z - s(t) = \mu \left( i + \frac{2}{\zeta + i}\right) , \end{aligned}$$

of a ring $R(t)< |\zeta | < 1$ in the complex $\zeta$-plane, $\zeta =-ir{e^{i\theta }}$, $-\pi<\theta <\pi$, onto the flow region $\Omega _2(t)$ in the complex *z*-plane, $z = x + iy$, see Fig. 12 in Appendix. A similar conformal mapping was used by Wang (2004)^46^. Here $\mu =\sqrt{{h^2} - 1}$ and $R=h-\mu$. Time *t* is a parameter in this subsection.

Equation (22) defines the relations $x=x(r,\theta ,t)$, $y=y(r,\theta ,t)$, $\alpha =\alpha (r,\theta ,t)$ and $\rho =\rho (r,\theta ,t)$ between the coordinates in the physical plane and the polar coordinates *r*, $\theta$ in the plane of the conformal mapping. The velocity potential in the coordinates *r*, $\theta$,

23 $$\begin{aligned} \phi (r,\theta ,t)=\varphi (x(r,\theta ,t),y(r,\theta ,t),t), \end{aligned}$$

can be decomposed as

24 $$\begin{aligned} \phi (r,\theta ,t)={\dot{s}}(t)\phi _1 (r,\theta ,h) - {\dot{h}}(t)\phi _2 (r,\theta ,h), \end{aligned}$$

see condition (20). The potentials $\phi _j(r,\theta ,h)$, $j=1,2$, are solutions of the following problems,

25 $$\begin{aligned} \left\{ \begin{aligned} \nabla ^2 \phi _j&= 0 \qquad \, (R<r<1),\\ \partial \phi _j/\partial r&=0 \qquad \, (r=1),\\ \partial \phi _j/\partial r&=f_j(\theta ) \,\, (r=R),\\ \phi _j(1,0,h)&=0, \end{aligned} \right. \end{aligned}$$

where

26 $$\begin{aligned} f_1(\theta )=\frac{2\mu }{R}\sum \limits _{n = 1}^\infty { nR^n\sin (n\theta )}, \,\, f_2(\theta )=-\frac{2\mu }{R}\sum \limits _{n = 1}^\infty { nR^n\cos (n\theta )}, \end{aligned}$$

see Appendix for details. The solutions of Eqs. (25) and (26) are

27 $$\begin{aligned} {\phi _1}(r,\theta ,h) = -2\mu \sum \limits _{n = 1}^\infty {\varphi _{n}(h) ({r^n} + {r^{ - n}})} \sin (n\theta ), \end{aligned}$$

28 $$\begin{aligned} {\phi _2}(r,\theta ,h) =2\mu \sum \limits _{n = 1}^\infty {\varphi _{n}(h)\left[ {({r^n} + {r^{ - n}})\cos (n\theta ) - 2} \right] }, \end{aligned}$$

where ${\varphi _{n}} = {{R^{2n}}}/{(1 - R^{2n})}$. In particular, on the boundary $y=0$, we have,

29 $$\begin{aligned} \varphi (x,0,t)=\phi (1,\theta ,t)=-4\mu \left[ {\dot{s}} \sum \limits _{n = 1}^\infty {\varphi _n(h) \sin (n\theta )} +{\dot{h}} \sum \limits _{n = 1}^\infty \varphi _n(h) [\cos (n\theta )-1] \right] , \end{aligned}$$

where *x* and $\theta$ are related by $({x - s(t)})/{\mu }= {\sin \theta }/{(1-\cos \theta )}$.

### Hydrodynamic pressure along the ice/water interface

The first term in the formula (17) for the hydrodynamic pressure along the ice/water interface represents the hydrostatic pressure and two other terms represent the dynamic component of the pressure $p_d(x,t)$, where

30 $$\begin{aligned} p_d(x,t)= -\frac{\partial \varphi }{\partial t}(x, 0, t)- \frac{1}{2} {\varphi _x}^2(x, 0, t), \end{aligned}$$

and $\varphi (x, 0, t)$ is given by (29) in the parametric form. To evaluate the derivatives in (30), we write (29) in the form $\phi (1,\theta ,t)=\varphi [x(1,\theta ,t),0,t]$ and differentiate this equality in time and $\theta$,

31 $$\begin{aligned} \phi _\theta =\varphi _x x_\theta ,\,\, \phi _t=\varphi _x x_t+\varphi _t \qquad (y=0). \end{aligned}$$

Equations (30) and (31) provide

32 $$\begin{aligned} p_d(x,t)=-\frac{\partial \phi }{\partial t}+\frac{\partial \phi }{\partial \theta }\frac{x_t}{x_\theta }-\frac{1}{2} \left( \frac{\partial \phi }{\partial \theta }\right) ^2\frac{1}{{x_\theta }^2}, \end{aligned}$$

where

33 $$\begin{aligned} \begin{aligned} x(1,\theta ,t)&=s(t)+\mu \frac{\sin \theta }{1-\cos \theta },\,\, x_t(1,\theta ,t)={\dot{s}}+\frac{h{\dot{h}}}{\mu }\frac{\sin \theta }{1-\cos \theta },\\ x_\theta (1,\theta ,t)&=-\frac{\mu }{1-\cos \theta }, \,\, \frac{\text{d}\varphi _n}{\text{d}t}=-\frac{2n{\dot{h}}}{\mu }\frac{R^{2n}}{(1-R^{2n})^2}, \frac{\text{d}\mu }{\text{d}t}=\frac{h{\dot{h}}}{\mu }. \end{aligned} \end{aligned}$$

Equations (29), (32) and (33) yield the dynamic component of the pressure along the ice/water interface in the leading-order as $\varepsilon \rightarrow 0$, once the functions *s*(*t*), *h*(*t*) and their first and second derivatives in time are known.

### Motion of the cylinder

The hydrodynamic force components, which are represented by the integrals in (8) and (9) with the pressure given by the Bernoulli equation (7) in the dimensionless variables, are convenient to be written in the form

34 $$\begin{aligned} F_x(t)=- \int \limits _0^{2\pi } {p(1,\alpha ,t)\cos \alpha \text{d}\alpha }= \frac{\text{d}}{\text{d}t} \int \limits _0^{2\pi } {\varphi (1,\alpha ,t)\cos \alpha \text{d}\alpha }, \end{aligned}$$

35 $$\begin{aligned} F_y(t)=-\int \limits _0^{2\pi } {p(1,\alpha ,t)\sin \alpha \text{d}\alpha }=\frac{\text{d}}{\text{d}t} \int \limits _0^{2\pi } {\varphi (1,\alpha ,t)\sin \alpha \text{d}\alpha }+ \frac{1}{2}\int \limits _{-\infty }^{\infty } {{\varphi ^2_x}(x,0,t)\text{d}x} +\frac{\pi }{{\textrm{Fr}}^{2}}, \end{aligned}$$

see Newman (2018)^50^. Formulas (34) and (35) are obtained from Eq. (90) in section 4.12 of the Newman’s book, where the stationary control surface $S_c$ consists of the interval $y=0$, $-R_0<x<R_0$ and the semi-circle $x^2+y^2=R^2_0$, $y<0$ with $R_0\rightarrow \infty$. The scale of the force components is $\rho _w aV^2$. Note that the presence of the rigid wall at $y=0$ yields a vertical positive force component given by the integral along this wall in (35). This force component appears also in the total vertical force $F_0(t)$ acting on the horizontal rigid wall. Integrating the hydrodynamic pressure (30) along the wall, we find

36 $$\begin{aligned} F_0(t)=-\frac{\text{d}}{\text{d}t} \int \limits _{-\infty }^{\infty }{{\varphi }(x,0,t)\text{d}x}-\frac{1}{2}\int \limits _{-\infty }^{\infty } {{\varphi ^2_x}(x,0,t)\text{d}x}. \end{aligned}$$

**Fig. 3:**
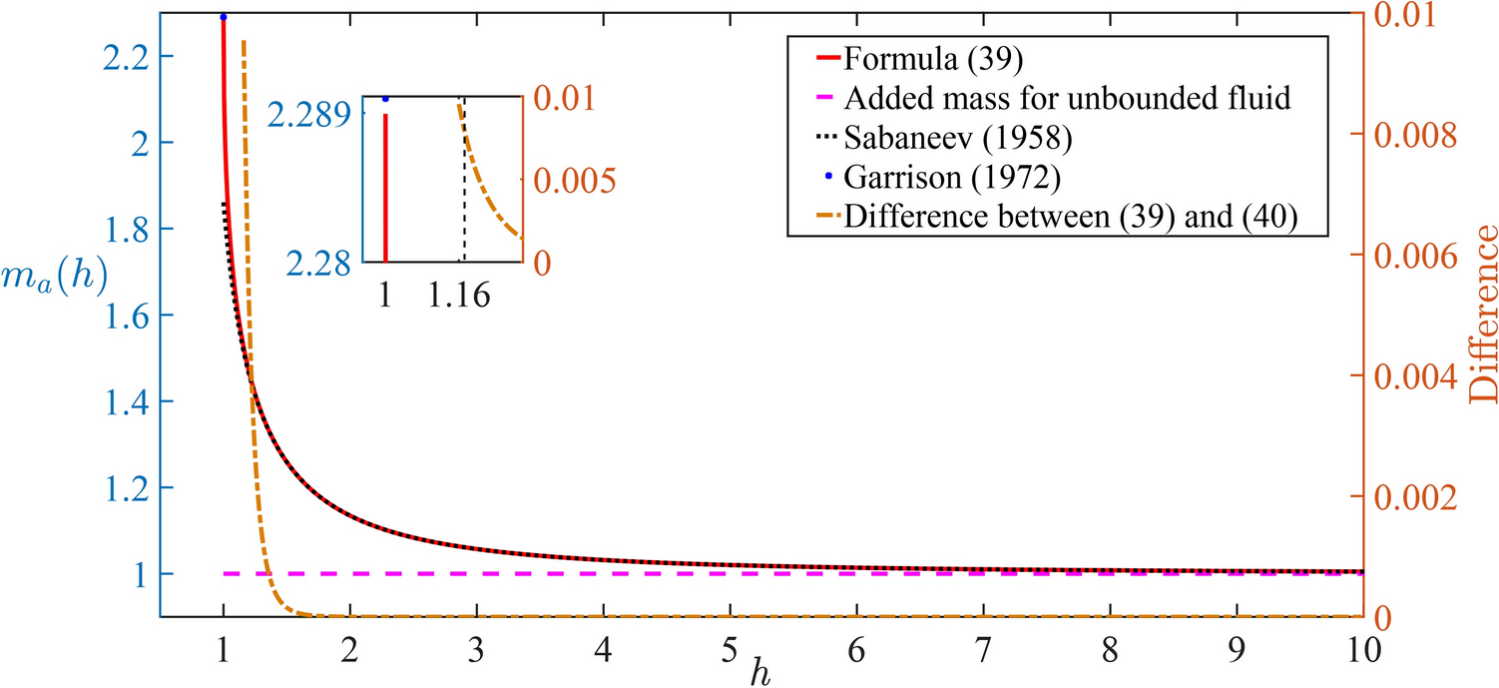
The dimensionless added mass of a circular cylinder near a rigid horizontal plate calculated by formula (39) (solid line), by Sabaneev (1958)^51^ (dotted line), by Garrison (1972)^54^ (point), in unbounded fluid (dashed line), together with the difference (dash-dotted line) between the numerical value (39) and the asymptotic formula (40).

Using the decomposition (24) in the physical plane, $\varphi (\rho ,\alpha ,t)={\dot{s}}(t)\varphi _1 (\rho ,\alpha ,h) - {\dot{h}}(t)\varphi _2 (\rho ,\alpha ,h)$, where $\varphi _1(\rho ,\alpha ,h)$ is an even function of $\alpha$ and $\varphi _2 (\rho ,\alpha ,h)$ is an odd function of $\alpha$, see (18)–(21), and the relations $\cos \alpha =\mu \sin \theta /{(h-\cos \theta )}$, $\sin \alpha =h-\mu ^2/{(h-\cos \theta )}$, we obtain

37 $$\begin{aligned} \int \limits _0^{2\pi } {\varphi (1,\alpha ,t)\cos \alpha \text{d}\alpha }={\dot{s}} \int \limits _0^{2\pi } {\varphi _1(1,\alpha ,t)\cos \alpha \text{d}\alpha }={\dot{s}} \int \limits _{-\pi }^{\pi } {\phi _1(R,\theta ,h) \frac{\mu ^2\sin \theta \text{d}\theta }{(h-\cos \theta )^2}} =-{\dot{s}} \pi m_a(h), \end{aligned}$$

38 $$\begin{aligned} \int \limits _0^{2\pi } {\varphi (1,\alpha ,t)\sin \alpha \text{d}\alpha }=-{\dot{h}} \int \limits _0^{2\pi } {\varphi _2(1,\alpha ,t)\sin \alpha \text{d}\alpha }={\dot{h}} \int \limits _{-\pi }^{\pi } {\phi _2(R,\theta ,h) \frac{\mu (h\cos \theta -1)}{(h-\cos \theta )^2}\text{d}\theta } ={\dot{h}} \pi m_a(h), \end{aligned}$$

where

39 $$\begin{aligned} m_a(h)=4 {\mu ^2}\sum \limits _{n = 1}^\infty nR^{2n}\frac{{1 + R^{2n}}}{{1 - R^{2n}}} \end{aligned}$$

is the dimensionless added mass of the circular cylinder moving under the rigid horizontal plate with the scale of the added mass being $\pi a^2 \rho _w$, see (8) and (9), which is the added mass of the circular cylinder moving in an unbounded fluid. The added mass $m_a(h)$ was calculated by Sabaneev (1958)^51^ using asymptotic methods,

40 $$\begin{aligned} m_a(h)= {1 + \frac{1}{2{h ^2}} + \frac{1}{{{2^3}{h ^4}}} + \frac{3}{{{2^5}{h ^6}}} + \frac{1}{{{2^4}{h ^8}}} + \frac{{23}}{{{2^9}{h ^{10}}}} + \frac{{71}}{{{2^{11}{h ^{12}}}}} + \cdots } \end{aligned}$$

in our notations, for $h\gg 1$. This formula was reproduced by Korotkin (2008)^52^ and Knight et al. (2024)^53^. The added mass $m_a(h)$ is shown in Fig. 3 together with the difference between the added mass provided by formula (39) and by the asymptotic formula (40) where seven terms are retained. It is seen that the difference is less than 0.01 for $h>1.16$. The added mass calculated by (39) for *h* being very close to but not equal to 1 gives $m_a=2.289$, which has a relative difference of $0.04\%$ from the value $m_a(1)=\pi ^2/3-1$ calculated by Garrison (1972)^54^. Note that $m_a(h)$ monotonically decreases with increase of the distance *h* from the plate.

The integral along the wall in (35) and (36) is evaluated using (29),

41 $$\begin{aligned} \int \limits _{-\infty }^{\infty } {{\varphi ^2_x}(x,0,t)\text{d}x}=2\pi ({\dot{s}}^2+{\dot{h}}^2)K(h),\, K(h)=8\mu \sum \limits _{n = 1}^\infty n \varphi _{n} \left( n \varphi _{n}-(n+1) \varphi _{n+1}\right) , \end{aligned}$$

where ${\varphi _{n}} (h)= {{R^{2n}}}/{(1 - R^{2n})}$, see (27) and (28). Detailed calculations leading to Eqs. (37)–(39) and (41) can be found in Appendix, where it is also shown that

42 $$\begin{aligned} K(h)=-\frac{1}{2}\frac{\text{d}m_a}{{\text{d}h}}. \end{aligned}$$

Equations (8), (9) and (34)–(42) provide

43 $$\begin{aligned} \frac{\text{d}}{{\text{d}t}}\left( [M+m_a(h)]\frac{\text{d}s}{{\text{d}t }} \right) = 0, \end{aligned}$$

44 $$\begin{aligned} \frac{\text{d}}{{\text{d}t}}\left( [M+m_a(h)] \frac{\text{d}h}{{\text{d}t }}\right) = \frac{1}{2} ({\dot{s}}^2+{\dot{h}}^2) \frac{\text{d}m_a}{{\text{d}h}}+\frac{M-1}{{\textrm{Fr}}^{2}}. \end{aligned}$$

Equations (43) and (44) can be integrated using the initial conditions (10) with the result

45 $$\begin{aligned} {\dot{s}}(t)=S(h)\cos \beta , \end{aligned}$$

46 $$\begin{aligned} {\dot{h}}^2(t)=S(h)[1-S(h)\cos ^2\beta +\delta (h-h_0)], \end{aligned}$$

where

47 $$\begin{aligned} S(h)=\frac{M+m_a(h_0)}{M+m_a(h)}, \,\, \delta =2\frac{M-1}{{{\textrm{Fr}}}^2[M+m_a(h_0)]}. \end{aligned}$$

For a heavy cylinder with its mass being greater than the added mass of the cylinder in unbounded fluid, $M>1,$ the parameter $\delta$ is positive. During the rising stage, when the cylinder approaches the rigid plate, the distance of the cylinder centre, *h*(*t*), from the plate monotonically decreases from $h_0$ either to 1, if the cylinder impacts the plate at the end of the stage, or to a certain distance $h_*>1$, where the cylinder stops, ${\dot{h}}(t)=0$, and moves downwards from the plate thereafter without contacting the plate.

We are concerned with the stages where $h<h_0$ and the hydrodynamic loads acting on the plate are highest. During this stage,we have $h-h_0<0$, which gives $m_a(h)>m_a(h_0)$, see Fig. 3. It further gives $0<S(h)<1,$ see Eq. (47). Therefore, $1-S(h)\cos ^2\beta >0$ and $\delta (h-h_0)$ in (46) is negative for $\delta >0$ and $h<h_0$. If $\delta <0,$ which is for a light cylinder, then ${\dot{h}}(t) <0$, the cylinder monotonically approaches the plate until its impact onto the plate when $h=1$ at the speed

48 $$\begin{aligned} |{\dot{h}}|=\sqrt{S(1)[1-S(1)\cos ^2\beta +\delta (1-h_0)]}. \end{aligned}$$

A heavy cylinder with $M>1$ and $\delta >0$ may impact the plate if $\delta$ is relatively small,

49 $$\begin{aligned} \delta < \frac{1-S(1)\cos ^2\beta }{h_0-1}, \end{aligned}$$

which implies high initial speed *V* of the cylinder,

50 $$\begin{aligned} \frac{V^2}{ga}={{\textrm{Fr}}}^2>2\frac{M-1}{M+m_a(h_0)}\frac{h_0-1}{1-S(1)\cos ^2\beta }. \end{aligned}$$

The inequality (50) can be presented for $\beta =\pi /2$ (strictly vertical motion of the cylinder) in the form

51 $$\begin{aligned} [{{\textrm{Fr}}^2}-2(h_0-1)]M>-[2(h_0-1)+m_a(h_0){{\textrm{Fr}}^2}], \end{aligned}$$

where the right-hand side is negative. It is convenient to introduce a critical Froude number ${{\textrm{Fr}}^*}=\sqrt{2(h_0-1)},$ which depends only on the initial submergence depth of the cylinder $h_0$. The inequality (51) shows that the impact of the cylinder onto the plate occurs for any mass of the cylinder *M* if ${{\textrm{Fr}}}>{{\textrm{Fr}}^*}$, and the mass of the cylinder should satisfy the inequality

52 $$\begin{aligned} M < \frac{ m_a(h_0)+({\textrm{Fr}}^*/{\textrm{Fr}})^2}{({\textrm{Fr}}^*/{\textrm{Fr}})^2-1}, \end{aligned}$$

for small initial speeds of the cylinder, ${{\textrm{Fr}}}<{{\textrm{Fr}}^*}$. Similar results can be obtained for other angles $\beta$ but only numerically due to dependence of *S*(1) on $h_0$ in (50).

**Fig. 4:**
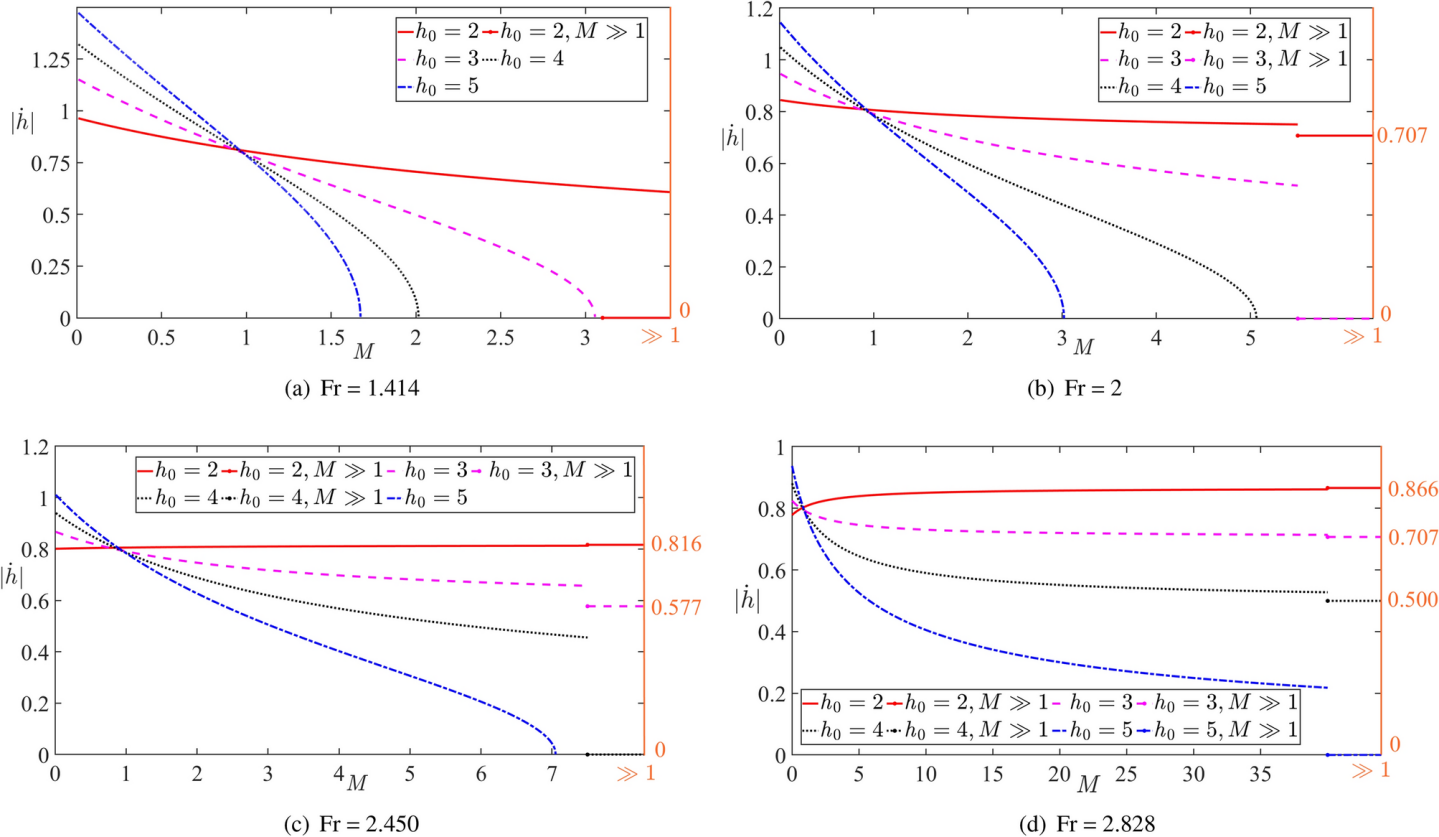
The dimensionless impact speed as a function of the dimensionless mass *M* of the cylinder for $\beta =\pi /2,$ $h_0=2$ (solid line), $h_0=3$ (dashed line), $h_0=4$ (dotted line) and $h_0=5$ (dash-dotted line) with different Froude numbers.

The dimensionless impact speed (48), which is the ratio of the dimensional impact speed to the initial speed *V* of the cylinder, is shown in Fig. 4 as a function of the dimensionless mass *M* of the cylinder for the vertical motion, $\beta =\pi /2$, and different initial positions of the cylinder, $h_0=2, 3, 4, 5$. The corresponding critical Froude numbers are ${{\textrm{Fr}}^*}=1.414, 2, 2.450, 2.828$. The calculations are performed for different initial speeds of the cylinder corresponding to ${{\textrm{Fr}}}=1.414, 2, 2.450$ and 2.828. Here the Froude numbers are equal to one of the critical Froude numbers of the selected $h_0$. Note that for very heavy cylinders, $M\gg 1$, Eqs. (47) and (48) give

53 $$\begin{aligned} S(h)\approx 1, \, \delta \approx \frac{2}{{{{\textrm{Fr}}}^2}}, \, |{\dot{h}}|=\sqrt{1-({\textrm{Fr}}^*/{\textrm{Fr}})^2} \end{aligned}$$

for ${{\textrm{Fr}}}>{{\textrm{Fr}}^*}$. For smaller initial speeds, ${{\textrm{Fr}}}<{{\textrm{Fr}}^*}$, the impact occurs only for relatively light cylinders such that the inequality (52) is satisfied. For a cylinder of zero mass, $M=0$, we find $S(1)=m_a(h_0)/m_a(1)$, $\delta = -2 {{\textrm{Fr}}}^{-2}/m_a(h_0)$ and

54 $$\begin{aligned} |{\dot{h}}|=\sqrt{\frac{m_a(h_0)}{m_a(1)} \left[ 1+\left( \frac{{\textrm{Fr}}^*}{ {\textrm{Fr}} } \right) ^2 \frac{1}{m_a(h_0)}\right] }. \end{aligned}$$

It is seen that the impact speed of such a cylinder can be rather high for large ${{\textrm{Fr}}^*}$, this is, for large initial submergence depth. Such a cylinder is accelerated towards the plate by the buoyancy force with negligible gravity force. Note that we do not account for viscous forces acting on the moving cylinder. For the conditions of Fig. 4, we can approximately take $m_a(h_0)\approx 1$ and $m_a(1)\approx 2.29$, see Fig. 3. It is seen that the impact speed (54) monotonically increases with increase of the submergence depth $h_0$, which is consistent with the experimental results by Ni et al. (2023)^38^, see Figure 13 there.

In Fig. 4a, the Froude number ${{\textrm{Fr}}}=V/\sqrt{ga}=1.414$ is the critical Froude number for $h_0=2$. The impact speed for $h_0=2$ as a function of the cylinder mass *M* is shown by the red line. The impact speed tends to zero as $M\rightarrow \infty$, see (53), and is slightly below 1 at $M=0$, see (54). For $h_0=3, 4$ and 5, the impact occurs only for *M* satisfying inequality (52). In Fig. 4b, the Froude number ${{\textrm{Fr}}}=2$ is critical for $h_0=3$ and supercritical for $h_0=2$, where the impact speed tends to the value given by (53) as $M\rightarrow \infty$. Impact occurs only for moderate masses of the cylinder for $h_0=4$ and 5. Figure 4d, where ${{\textrm{Fr}}}=2.828$ is the critical Froude number for $h_0=5$, shows that impact occurs for any selected submergences $h_0$.

Just before the impact, when the cylinder is very close to the ice plate, the elastic deformations of the plate can be important near the impact place. These elastic deflections should be included in the model of ice plate interaction with the free moving cylinder. The problems of impact between a cylinder and a floating ice plate can be studied using either the Hertz theory^55^ or a Winkler elastic layer approximation for the lower surface of the plate^56^. The Hertz theory was used in the problem of a rigid body impact onto an Euler-Bernoulli beam by Timoshenko and Young (1955)^57^. The model of Winkler elastic layer on the impacted surface of a floating ice plate was used recently by Khabakhasheva et al. (2018)^58^, Khabakhasheva and Korobkin (2021)^59^.

In the present study, only the motions of a cylinder under the plate without impacts with the plate are considered. This implies that the cylinder approaches the plate from below, then stops at a certain submergence $h_*$ and goes downwards from the plate thereafter. The value $h_*$ is calculated using that the condition ${\dot{h}}(t)=0$ at $h=h_*$ in (46),

55 $$\begin{aligned} 1-S(h_*)\cos ^2\beta -\delta (h_0-h_*)=0. \end{aligned}$$

Equation (55) has a solution only for $\delta >0,$ which is for $M>1$. Note that $h_*$ should be greater than one. For $\beta =\pi /2$, Eq. (55) gives $h_*=h_0-1/\delta$ and the condition $h_*>1$ implies

56 $$\begin{aligned} {{\textrm{Fr}}}^2 < \frac{2h_0(M-1)}{M+m_a(h_0)}. \end{aligned}$$

The time $t_*$ required by the cylinder to reach the distance $h_*$ is obtained by integrating (46) in time,

57 $$\begin{aligned} t_*=\int ^{h_0}_{h_*} \frac{\textrm{d}h}{\sqrt{S(h)[1-S(h)\cos ^2\beta -\delta (h_0-h)]}}. \end{aligned}$$

We shall calculate the deflection of the ice plate and strains in the plate caused by the hydrodynamic pressure (30).

### Deflection of the ice cover

In the leading order approximation, the ice deflection, *w*(*x*, *t*), is governed by the thin elastic plate equation, see (11), (7) and (30)

58 $$\begin{aligned} {\chi \varepsilon }\frac{\partial ^2 w}{\partial t^2} + \frac{\partial ^4 w}{\partial x^4}+\frac{\varepsilon }{{\textrm{Fr}}^{2}} w= p_d(x, t) (-\infty<x<\infty ), \end{aligned}$$

where the dynamic component of the pressure, $p_d(x, t)$, is calculated using (24), (27), (28), (32) and (33). Eq. (58) is to be solved subject to the initial conditions (12) and the far-field condition (13). This problem is solved using the Fourier transform in the *x*-direction,

59 $$\begin{aligned} {w^F}\left( {k,t} \right) = \int \limits _{ - \infty }^\infty {w\left( {x,t} \right) {e^{ - ikx}}} {\text {d}}x,\,\, w\left( {x,t} \right) = \frac{1}{{{2\pi } }}\int \limits _{ - \infty }^\infty {{w^F}\left( {k,t} \right) {e^{ikx}}} {\text {d}}k. \end{aligned}$$

The plate equation (58) provides,

60 $$\begin{aligned} \chi \varepsilon w_{tt}^F + \left( {{k^4} + \varepsilon {{\textrm{Fr}}^{-2}} } \right) {w^F} = {p^F_d}(k,t). \end{aligned}$$

The general solution of the linear differential equation with constant coefficients (60) reads

61 $$\begin{aligned} {w^F}(k,t) = {c_1}(k)\sin (\omega t) + {c_2}(k)\cos (\omega t) + \frac{1}{{\omega \chi \varepsilon }}\int \limits _0^t {{p^F_d}(k,\tau )\sin \left[ {\omega \left( {t - \tau } \right) } \right] } \textrm{d}\tau , \end{aligned}$$

where $\omega ^2=({{{k^4} + \varepsilon {{\textrm{Fr}}^{-2}} }})/({\chi \varepsilon })$. The initial condition (12) yield $c_1(k)=c_2(k)=0$. Then

62 $$\begin{aligned} {w^F}(k,t) =\frac{1}{{ \chi \varepsilon \omega (k)}} \sin (\omega t) I^c(k,t) -\frac{1}{{ \chi \varepsilon \omega (k) }} \cos (\omega t) I^s(k,t), \end{aligned}$$

where

63 $$\begin{aligned} I^c(k,t)=\int \limits _0^t {{p^F_d}(k,\tau )\cos (\omega \tau ) }\textrm{d}\tau ,\, I^s(k,t)=\int \limits _0^t {{p^F_d}(k,\tau )\sin (\omega \tau ) }\textrm{d}\tau . \end{aligned}$$

The integrals (63) are evaluated by solving the differential equations

64 $$\begin{aligned} \frac{\text{d}I^c}{\text{d}t}={p^F_d}(k,t) \cos [\omega (k) t],\, \frac{\text{d}I^s}{\text{d}t}={p^F_d}(k,t) \sin [\omega (k) t], \end{aligned}$$

subject to the initial conditions $I^c(k,0)=I^s(k,0)=0$, where *k* is a parameter.

Therefore, the ice deflection in the leading order is given by

65 $$\begin{aligned} w\left( {x,t} \right) = \frac{1}{{2\pi \chi \varepsilon }}\int \limits _{ - \infty }^\infty {\frac{{{e^{ikx}}}}{{\omega (k)}} \left( {\sin (\omega t){I^c}(k,t) - \cos (\omega t){I^s}(k,t)} \right) } \textrm{d}k, \end{aligned}$$

the speed of the deflection by

66 $$\begin{aligned} {w_t}\left( {x,t} \right) = \frac{1}{{2\pi \chi \varepsilon }}\int \limits _{ - \infty }^\infty {\left( {\cos (\omega t){I^c}(k,t) + \sin (\omega t){I^s}(k,t)} \right) {e^{ikx}}} \textrm{d}k, \end{aligned}$$

and the strains distributed along the upper surface of the ice by

67 $$\begin{aligned} {\epsilon }(x,t)= \frac{{\epsilon _{sc}}}{{2\pi \chi \varepsilon }}\int \limits _{ - \infty }^\infty {\frac{{{k^2}{e^{ikx}}}}{{\omega (k)}}\left( {\sin (\omega t){I^c}(k,t) - \cos (\omega t){I^s}(k,t)} \right) } \textrm{d}k, \end{aligned}$$

see (14).

Equation (64) are integrated in time using the fourth-order Runge-Kutta scheme, where $k=-200+0.01\times (j-1)$, $1\le j \le 40001$. This is, the infinite range of the Fourier parameter *k* is limited to the interval $[-200,200]$, which is divided into subintervals of length 0.01. The time step $\Delta t$ of integration depends on the pressure distribution and the frequency $\omega (k)$. A conservative estimate suggest $\omega (k) \Delta t < \pi /180$, which implies that the increment of the product $\omega t$ is selected to be smaller than $1^{\circ }$ at each time step. Note that, in the formula for $\omega (k)$, both $\chi$ and $\varepsilon$ are small. This implies a small time step $\Delta t$. Integrations with respect to *k* in (65)–(67) are performed by the trapezoidal rule with step $\Delta k=0.01$ for *x* from the interval $[-200,200]$ with the step $\Delta x=0.01$. Note that all equations are in the dimensionless variables. Convergence of the solution with respect to the interval of integration, step of integration and the time step is investigated in each case separately.

To justify the present numerical algorithm, we mimic the dynamic pressure distribution $p_d(x,t)$ in (58) as

68 $$\begin{aligned} p_d(x,t) = \frac{{ \mu (t) }}{{{x^2} + {\mu ^2(t) }}}, \end{aligned}$$

where $\mu (t)=\sqrt{h^2(t)-1}$ and *h*(*t*) is a fictitious distance in this example. We select $\mu =b+ct$, where $b>0$ and $c<0$ are constant parameters. This corresponds to the motion as $h(t)=\sqrt{\mu ^2(t)+1}$ with $h(0)=\sqrt{b^2+1}$. We find ${p^F_d}(k,t) = \pi {e^{ - \left| k \right| \mu (t)}}$. The formula (62) provides

69 $$\begin{aligned} {w^F}(k,t) = \frac{1}{\chi \varepsilon } \pi {e^{ - \left| k \right| b}}\frac{1}{{{ k ^2}{c^2} + {\omega ^2}}}\left( {{e^{ - \left| k \right| ct}} + \frac{1}{\omega }\left| k \right| c\sin \left( {\omega t} \right) - \cos \left( {\omega t} \right) } \right) . \end{aligned}$$

Correspondingly, the deflection *w*(*x*, *t*), the speed $w_t(x,t)$, and the strain $\epsilon (x,t)$ for this example are given by

70 $$\begin{aligned} w (x,t)= \frac{1}{{\chi \varepsilon }}\int \limits _0^\infty {{e^{ - kb}}\frac{{\cos \left( {kx} \right) }}{{{k^2}{c^2} + {\omega ^2}}}\left( {{e^{ - kct}} + \frac{1}{\omega }kc\sin \left( {\omega t} \right) - \cos \left( {\omega t} \right) } \right) \textrm{d}k}. \end{aligned}$$

71 $$\begin{aligned} {w_t}(x,t) = \frac{1}{{\chi \varepsilon }} \int \limits _0^\infty { {{{e^{ - kb}}}} \frac{{\cos \left( {kx} \right) }}{{{k^2}{c^2} + {\omega ^2}}}\left( { - kc{e^{ - kct}} + kc\cos \left( {\omega t} \right) + \omega \sin \left( {\omega t} \right) } \right) \textrm{d}k}, \end{aligned}$$

72 $$\begin{aligned} {\epsilon }(x,t)= \frac{{\epsilon _{sc}}}{{\chi \varepsilon }} \int \limits _0^\infty { {k^2}{e^{ - kb}}\frac{{\cos \left( {kx} \right) }}{{{k^2}{c^2} + {\omega ^2}}}\left( {{e^{ - kct}} + \frac{1}{\omega }kc\sin \left( {\omega t} \right) - \cos \left( {\omega t} \right) } \right) \textrm{d}k}. \end{aligned}$$

To compare the analytical solutions (70)–(72) with the numerical solution of the plate equation (58), where the Fourier transforms and the Eq. (64) are integrated numerically, we select $b=1$, $c=-100$, ${\textrm{Fr}}=2$, and the parameters listed in (2), which gives $\chi =0.37$, $\varepsilon =2.0\times 10^{-4}$, $w_{sc}=1.0\times 10^{-4}\,\textrm{m}$, ${{\epsilon _{sc}}}=4.0\times 10^{-5}$. The dimensionless time step of the integration is taken as $\triangle t=0.0001$ for the selected parameters. It is shown that calculations with $\triangle t=0.0002$ provide the results the relative difference of which from the results with $\triangle t=0.0001$ does not exceed $2.6\times 10^{-4}\%$ for the deflection, $8.5\times 10^{-5} \%$ for the speed of the deflection, and $5.5\times 10^{-3}\%$ for the strains in the interval $-200\le x \le 200$. The relative difference $\triangle w_{rel}(t)$ of the deflections, for example, is calculated by the formula

73 $$\begin{aligned} \triangle w_{rel}(t)= 100\% \frac{{\mathop {\max }\limits _{ - 200 \le x \le 200} \left| {w(x,t,\Delta t = 0.0002) - w(x,t,\Delta t = 0.0001)} \right| }}{{\mathop {\max }\limits _{ - 200 \le x \le 200} \left| {w(x,t,\Delta t = 0.0001)} \right| }}. \end{aligned}$$

Note that the conservative estimate $\omega (k) \bigtriangleup t < \pi /180$ provides $\bigtriangleup t=3.7 \times 10^{-9}$ for $k=\pm 200$. We conclude that the integration of (64) in time with much larger time step provides accurate results.

**Fig. 5:**
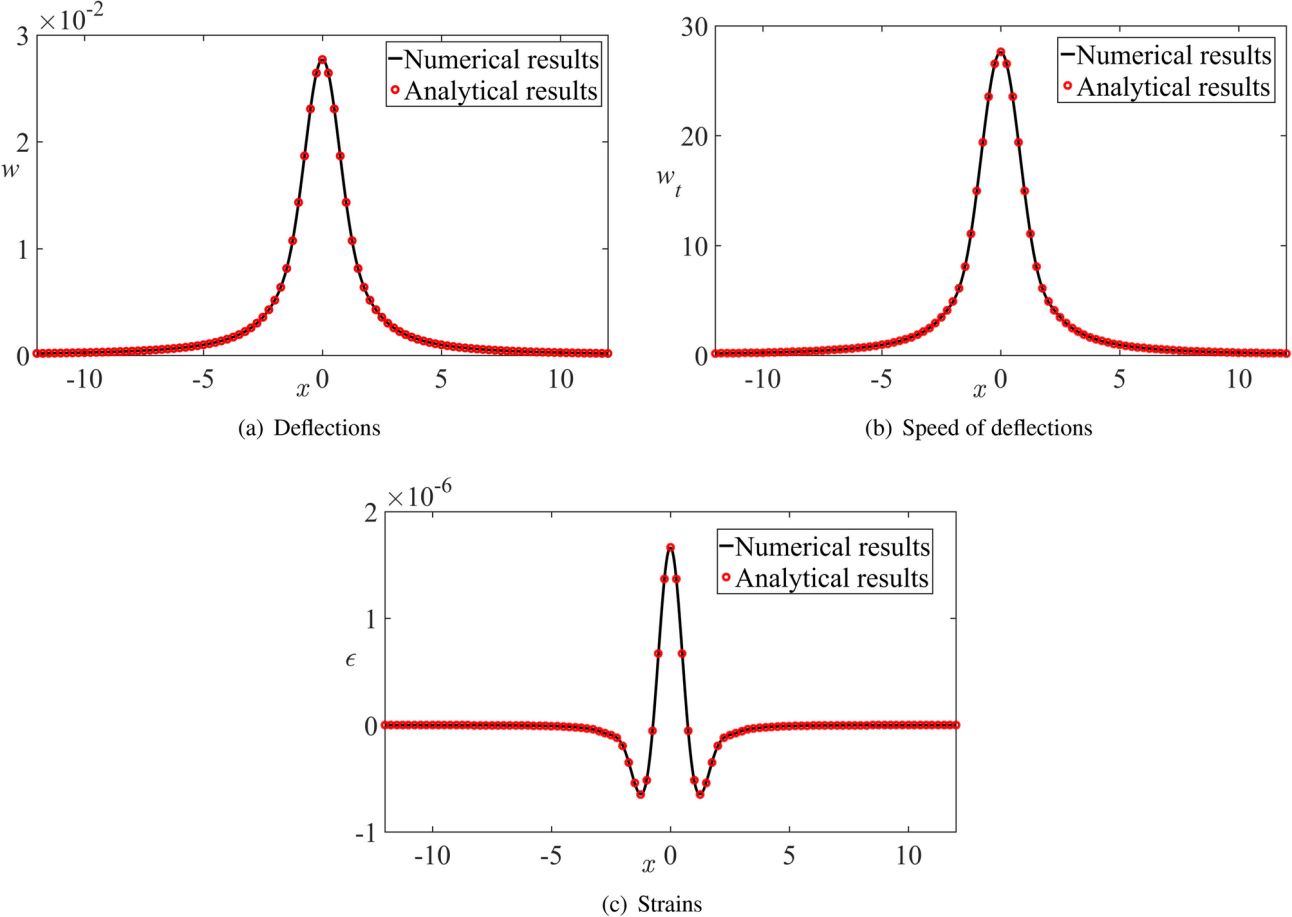
The comparison of the analytical results (70)–(72) (red circles) and numerical results (solid lines) in the dimensionless variables at the dimensionless time 0.002 with a time step of 0.0001 for (**a**) deflection, (**b**) speed of the deflection, and (**c**) strains. The relative difference between the analytical and numerical results does not exceed $0.033\%$ for the strains distributed along the upper surface of the plate.

The analytical (70)–(72) and the corresponding numerical results in the dimensionless variables are shown in Fig. 5 for dimensionless time $t=0.002$, corresponding to $\mu =0.8$. It is seen that the numerical algorithm of this section accurately describes deflections of the elastic plate and the strains in it. The presented analysis justifies the selected algorithm of calculation of the ice response.

## Results

### Free motion of the cylinder under the ice

The added mass $m_a(h)$ of the cylinder in the dimensionless variables is equal to 1 in unbounded fluid. The governing equations of the motion (43), (44) and the initial conditions (10) provide

74 $$\begin{aligned} s_u(t)=s_0+\cos \beta t, \end{aligned}$$

75 $$\begin{aligned} h_u(t)=h_0+\frac{M-1}{M+m_a}\frac{t^2}{2{{\textrm{Fr}}^{2}}}-\sin \beta t, \end{aligned}$$

for the motion of the cylinder in an unbounded fluid with account for gravity. The values for parameters listed in (2) and the mass ratio $M=2$ are used here. The initial position of the cylinder is selected as $s_0=0$, $h_0=2$. It can be obtained from Eq. (58) that the impact happens if the Froude number is larger than 0.799 for $\beta =\pi /2$ and 1.003 for $\beta =\pi /4$. We select $Fr=0.79$ and $\beta =\pi /4$ to avoid the impact between the cylinder and the plate.

**Fig. 6:**
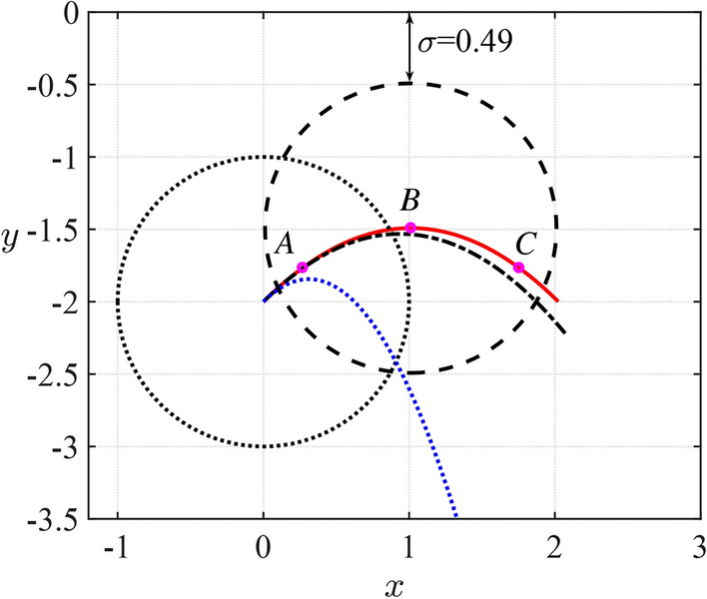
Dimensionless trajectories of the circular cylinder under a rigid ice plate (red solid line), in unbounded fluid (black dash-solid line) and in the air (blue dotted line). The dotted circle represents the initial position of the cylinder and the dashed circle shows the position of the cylinder closest to the ice plate ($M=2$, $s_0=0$, $h_0=2$, $Fr=0.79$ and $\beta =\pi /4$).

The trajectory of the cylinder predicted by (45) and (46) for its motion in a fluid under a rigid plane is shown in Fig. 6 together with the trajectories of the same cylinder moving in unbounded fluid and in the air. It should be noted that the density of the fluid enters the formula for *h*(*t*) only through the dimensionless mass *M*. The blue dotted line in Fig. 6 is for the same cylinder in the air. This trajectory is given by (74), (75), where the dimensionless mass of the cylinder $M^{(a)}=M'/(\pi a^2 \rho _a)=M\rho _w/\rho _a$, $\rho _a$ is the air density. Therefore, $M^{(a)}\gg M$. The difference between the distance of the cylinder centre from the ice $h_u(t)$ given by (75) and the distance $h_a(t)$ in the air is equal to

76 $$\begin{aligned} h_u(t)-h_a(t)=(\frac{M-1}{M+m_a}-\frac{M^{(a)}-1}{M^{(a)}+m_a})\frac{t^2}{2{{\textrm{Fr}}^{2}}} =-\frac{(M^{(a)}-M)(1+m_a)}{(M+m_a)(M^{(a)}+m_a)}\frac{t^2}{2{{\textrm{Fr}}^{2}}}. \end{aligned}$$

Therefore, the trajectory of the cylinder in the air is always below the trajectory of the same cylinder in water, $h_a(t)>h_u(t)$, as it is depicted in Fig. 6.

**Fig. 7:**
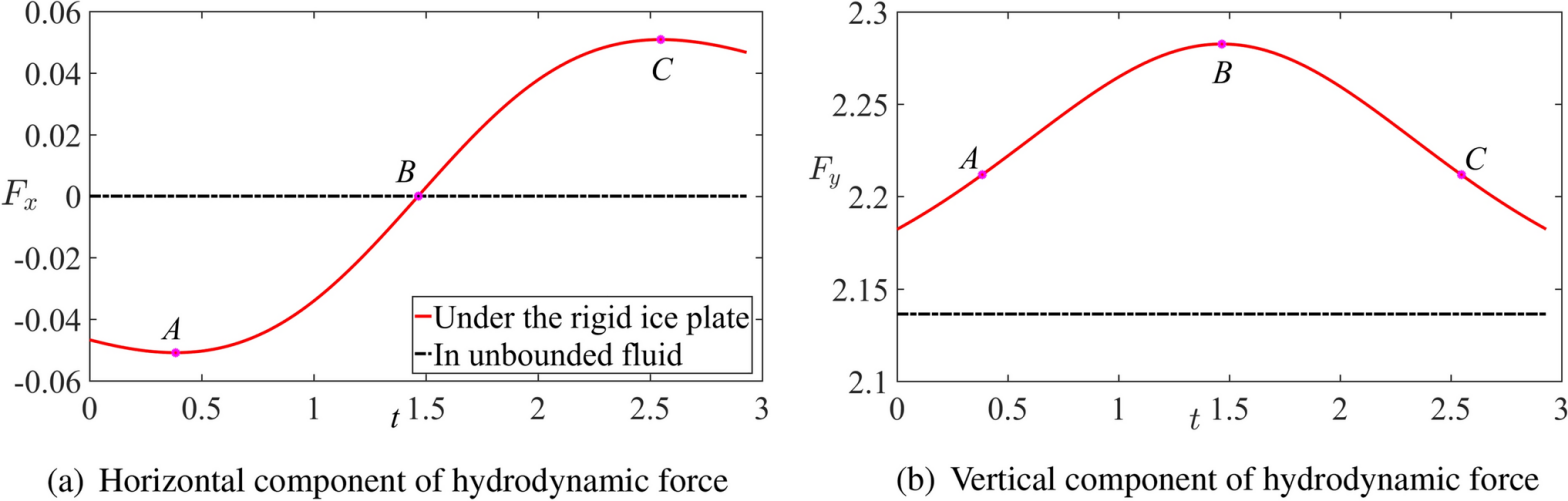
Dimensionless hydrodynamic force acting on the cylinder during its inertial motion under the rigid ice plate and in unbounded fluid ($M=2$, $s_0=0$, $h_0=2$, $Fr=0.79$ and $\beta =\pi /4$).

It is convenient to introduce a parameter $\sigma$ representing the dimensionless distance between the top of the cylinder and the rigid plate, $y=0$, where $\sigma =h-1$. The smallest distances during free motions of the cylinder corresponding to point *B*, in the three cases of Fig. 6, are $\sigma =0.49$ for motion under the plate, $\sigma =0.53$ for motion in the unbounded fluid and ${\sigma =0.84}$ for motion without hydrodynamic forces acting on the cylinder. This result confirms that there is an attraction between the rigid boundary and the cylinder, which is also clear from Fig. 7b where the vertical component of the hydrodynamic force is shown as a function of time. The force components $F_y(t)$ and $F_x(t)$ are given by the formula

77 $$\begin{aligned} F_y=\frac{M S(h)}{2(M+m_a)} \frac{\textrm{d}m_a(h) }{\textrm{d}h} \left[ 1-\delta (h_0-h)-2S(h)\cos ^2\beta \right] +\frac{m_a+1}{m_a+M} \frac{M}{{\textrm{Fr}}^2}, \end{aligned}$$

78 $$\begin{aligned} F_x=-\frac{M S(h)}{M+m_a(h)} \frac{\textrm{d}m_a(h) }{\textrm{d}h} \frac{\textrm{d}h}{\textrm{d}t }\cos \beta , \end{aligned}$$

where ${\dot{h}}(t)$ is given by (46) with account for its sign. It is seen that the dimensionless vertical force component is positive and peaks when the cylinder is the most close to the plate. The presence of the plate increases the vertical force compared with the force in the unbounded fluid, which is shown by the dashed line in Fig. 7b.

The horizontal dimensionless component $F_x(t)$ of the hydrodynamic force acting on the cylinder is zero in unbounded fluid because $\textrm{d}m_a(h) /\textrm{d}h=0$. For a cylinder moving under the plate, this force component decelerates the cylinder before the cylinder reaches its highest point, see Fig. 7a, and accelerates the cylinder in the horizontal direction thereafter. The points *A* and *C* in Figs. 6 and 7 correspond to the minimum and maximum of the force component $F_x(t)$, respectively.

### Ice response caused by inertial motion of a submerged cylinder

The obtained results are presented in terms of the pressure distributed along the ice/water interface, ice deflection, speed of deflection and strain distribution along the upper surface of the ice due to an inertial motion of the circular cylinder as it is described in last section. The calculations are performed for the ice sheet of thickness $h_i=20\, \textrm{cm}$, Young modulus $E=4.2\times 10^9 \,\mathrm{N/m}^2$, Poisson ratio $\nu =0.3$ and density of ice $\rho _i=917\, \mathrm{kg/m}^3$. The cylinder of radius $a=50\, \textrm{cm}$ starts its motion at a certain depth under the ice with speed $V=1.75 \,\mathrm{m/s}$, see conditions (2). Then the rigidity of the ice plate $D = {{E{h_i}^3} / {\left[ {12(1 - {\nu ^2})} \right] }}=3.077\cdot 10^{6} \,\textrm{N} \cdot \textrm{m}$ implies the deflection scale $w_{sc}=\rho _w V^2 a^4 /D$, where $\rho _w=1000 \, \mathrm{kg/m}^3$ is the water density, being $w_{sc}=6.22 \cdot 10^{-5} \,\textrm{m}$. The dynamic pressure scale is $3062.5\,\textrm{Pa}$, which is much smaller than the ambient atmospheric pressure of $101,325 \,\textrm{Pa}$. The time scale *a*/*V* is $0.28\,\textrm{s}$ and the length scale is $0.5\, \textrm{m}$. The scale of the deflection speed, $w_{sc}/(a/V)$, is equal to $2.22 \cdot 10^{-4} \, \mathrm{m/s}$ for the conditions under consideration. All calculations are performed in the dimensionless variables.

The intervals of integration in the direct and inverse Fourier transforms (59), step of the integration and the time step of integration in (63) are chosen as $[-200,200]$, 0.01 and 0.002, respectively, to ensure convergence and good accuracy of the solution. Three time instances corresponding to the points *A*, *B* and *C* in Fig. 6 including the highest position of the cylinder at $t=1.466$, and two extremum points of $F_x(t)$, see Fig. 7a, at $t=0.384$ and 2.548 are selected here to show the dynamic component of the hydrodynamic pressure, deflection, speed of deflection and strain distributed along the ice. The conditions of the motion are $M=2$, $s_0=0$, $h_0=2$, $Fr=0.79$ and $\beta =\pi /4$.

**Fig. 8:**
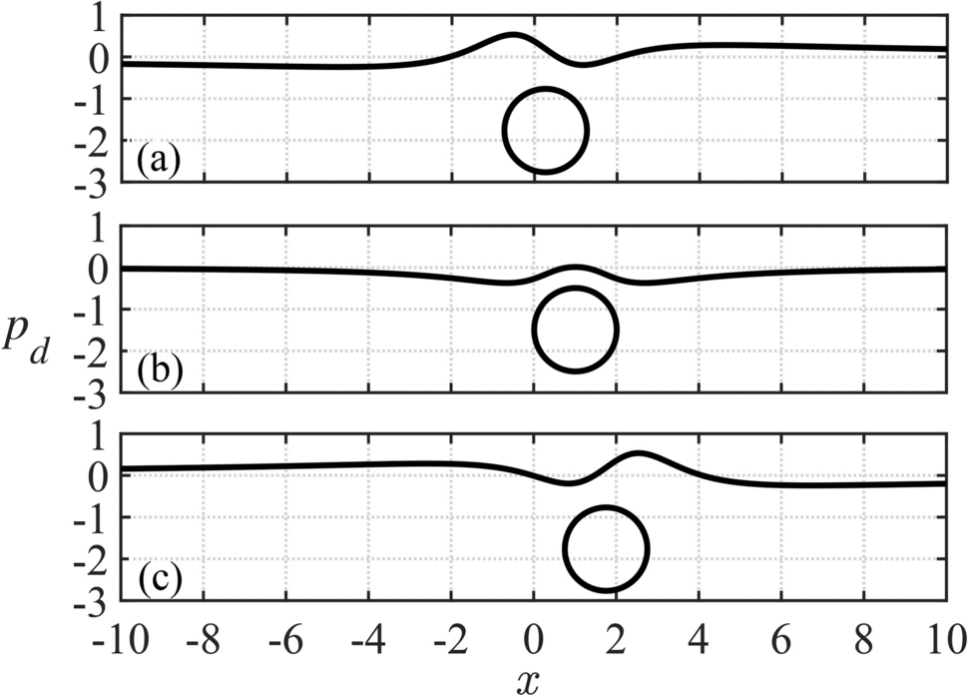
Dimensionless dynamic component of the hydrodynamic pressure along the ice/water interface at (**a**) $t=0.384$, (**b**) $t=1.466$ and (**c**) $t=2.548$ (points *A*, *B*, *C* in Fig. 6 respectively), with the corresponding positions of the cylinder ($M=2$, $s_0=0$, $h_0=2$, $Fr=0.79$, $\beta =\pi /4$, $a=0.5\, \textrm{m}$ and pressure scale is $3062.5\,\textrm{Pa}$ ).

Figure 8 shows the dimensionless dynamic component of the hydrodynamic pressure as a function of the dimensionless coordinate *x* along the ice/water interface at the time instants corresponding to the positions of the cylinder centre at the points *A*, *B*, *C* shown in Fig. 6. The vertical axis in Fig. 8 is for the dimensionless dynamic pressure. The pressure scale is 3062.5 Pa. The positions of the cylinder at these time instants are also shown. Comparing Fig. 8a,b, it can be noticed that the pressure decreases at the end of the rising stage (from point *A* to *B*) due to the decrease of the vertical speed of the cylinder. The maximum pressure occurs behind the cylinder during the rising stage (from point *A* to *B*) and in front of the cylinder during the falling stage (from point *B* to *C*). The pressure distribution is approximately symmetric with the vertical axis going through the center of the cylinder when the cylinder is at its highest point, *B*, as shown in Fig. 8b. We conclude that the pressure is not maximum for the highest position of the cylinder.

**Fig. 9:**
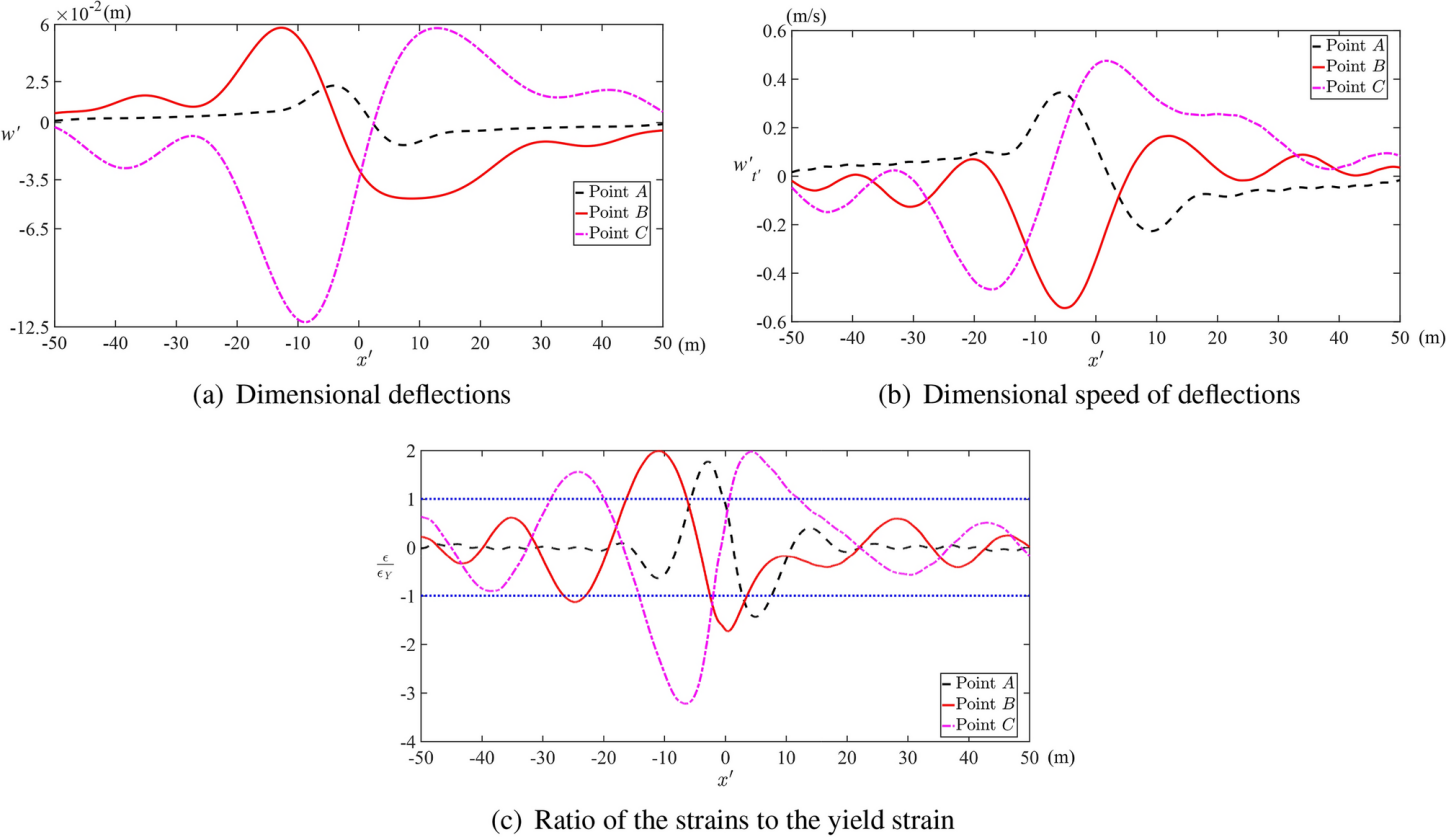
Dimensional (**a**) deflections, (**b**) speed of deflections, and (**c**) ratio of the strains distributed along the upper surface to the yield strain at the time instants corresponding to the points *A*, *B*, *C* in Fig. 6 for $M=2$, $s_0=0$, $h_0=2$, $Fr=0.79$, $\beta =\pi /4$, $\varepsilon =1.24 \times 10^{-4}$, $\epsilon _{Y}=8\times 10^{-5}$.

**Fig. 10:**
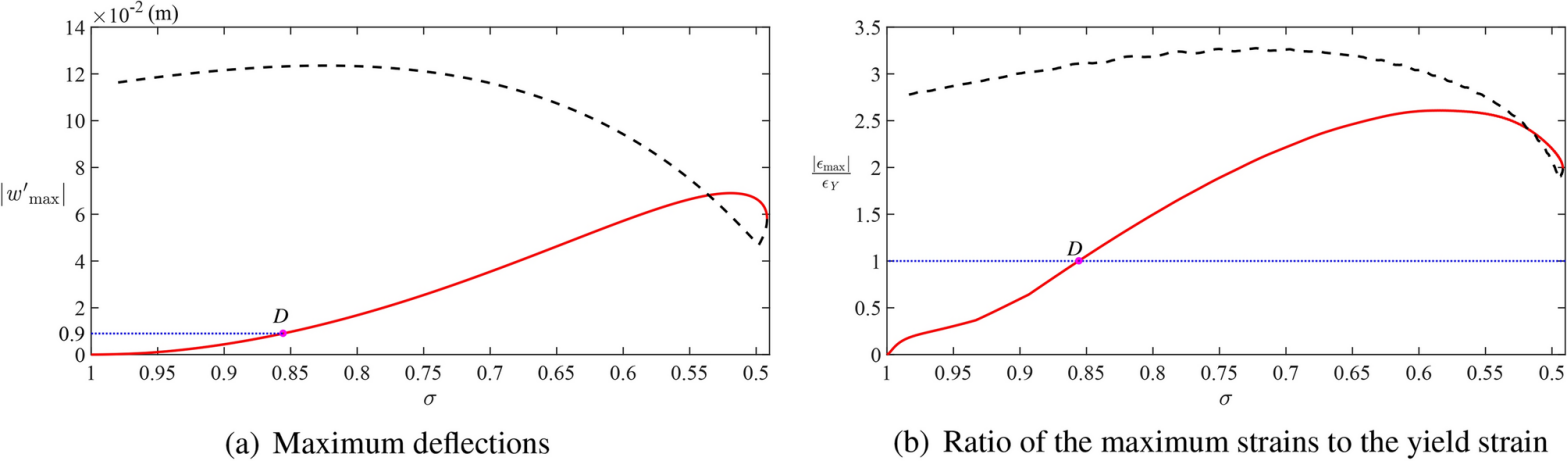
Dimensional (**a**) maximum deflections, and (**b**) ratio of the maximum strains distributed along the upper surface to the yield strain with respect to the distance $\sigma$ during the rising stage (red solid line) and falling stage (black dashed line) for $M=2$, $s_0=0$, $h_0=2$, $Fr=0.79$, $\beta =\pi /4$, $\varepsilon =1.24 \times 10^{-4}$, and $\epsilon _{Y}=8\times 10^{-5}$.

Figure 9 depicts the dimensional deflection, speed of deflection, and the strain distributions along the upper ice surface at the time instants corresponding to the points *A*, *B*, *C* in Fig. 6. The maximum dimensional deflection does not exceed 12.5 cm, which is smaller than the linear scale of the problem. Figure 10b predicts breaking the ice at very early stage where the maximum deflection 0.9 cm is much smaller than the radius of the cylinder, corresponding to point *D* in Fig. 10. Figure 9b shows that the speed of the deflections is smaller than the speed of the cylinder *V*. The maximum magnitude of the speed of the deflection is approximately 0.5 m/s in this case, which is more than three times smaller than the cylinder speed $V=$ 1.75 m/s. Therefore, the decoupled approach and linear dynamics of the ice plate are justified for these conditions. It would be helpful to solve this problem using the coupled approach and nonlinear theories of both the ice response and the flow under the ice and then compare the obtained results with the present ones. The coupled nonlinear problem is not considered in this paper. The ratio of the strain on the upper surface of the ice plate to the yield strain $\epsilon _{Y}=8\times 10^{-5}$ of the ice is shown in Fig. 9c. It is seen that the ice can be broken even before the cylinder arrives at position closest to the plate. See also Fig. 10b, where the maximum along the plate strain is shown for different $\sigma$ of the cylinder from the plate. The absolute maximum strain is achieved well before the cylinder arrives at its highest position, and the strains are greater at the stage, when the cylinder goes downwards from the plate. This result is conditional because the Fig. 10b predicts breaking the ice at a very early stage when the distance between the ice plate and the top of the cylinder is about 0.856*a* in the dimensional variables.

Figure 10a shows that the maximum along the ice plate deflection monotonically increases, when the cylinder approaches the ice plate, then it is reduced when the speed of the cylinder is small, and then the deflection increases again monotonically when the cylinder goes downwards from the plate.

**Fig. 11:**
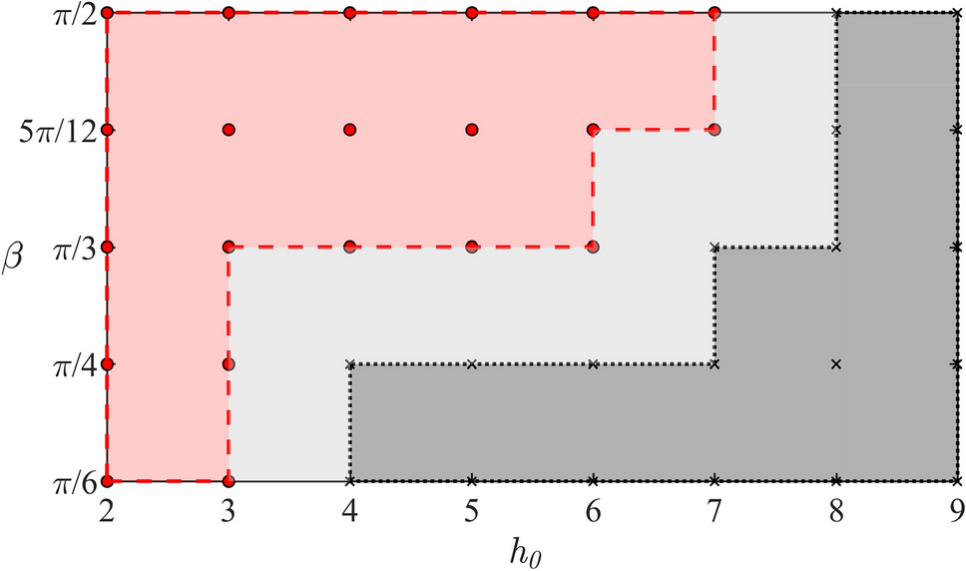
Initial conditions of the cylinder motion leading to the ice plate breaking, ${\epsilon >\epsilon _{Y}}$ during the motion, are shown by red circle markers, and the conditions where ${\epsilon <\epsilon _{Y}}$ during the motions are shown by cross markers ($M=2$, $Fr=0.79$).

If the radius of the cylinder, *a*, the mass of the cylinder $M'$ and the initial velocity *V* are prescribed, then the ice response depends only on the angle of attack $\beta$ and the dimensionless submergence depth $h_0$. It is clear that increasing $h_0$ and/or reducing the angle $\beta$ lead to decrease of the strains in the ice plate. The initial *x*-coordinate $s_0$ of the cylinder does not affect the ice response for isotropic ice plate of constant thickness. Calculations are performed for $h_0=j$, $2\le j \le 9$ and $\beta =\frac{\pi }{6}$, $\frac{\pi }{4}$, $\frac{\pi }{3}$, $\frac{5\pi }{12}$, $\frac{\pi }{2}$. The results are presented in Fig. 11. They imply that for given size of the cylinder and its initial kinetic energy the ice is broken only if the cylinder is placed initially close to the plate. The cylinder does not impact the ice for the selected conditions.

## Conclusion

Two-dimensional unsteady problem of an oblique inertial motion of a rigid circular cylinder beneath an ice cover and the response of the ice to this motion have been investigated. The ice sheet floating on water surface was modelled as a thin elastic plate of constant thickness and of infinite extent. The liquid under the ice was inviscid, incompressible and of infinite depth. The liquid flow caused by the cylinder motion was assumed potential. The motion of the cylinder is governed by its inertia, gravity, hydrodynamic force, deflection of the ice cover and the initial conditions. This problem of hydroelasticity was approximately decoupled for relatively small speeds of the body, see inequality (1).

Within the decoupled approach, the cylinder motion, the generated flow, and the hydrodynamic pressure in the fluid were determined without account for the ice deflection. The obtained pressure on the ice/water interface was applied to the ice plate, where the coupling between the ice plate deflection and the flow was retained only through the hydrostatic pressure. The inertia of the ice plate was also retained to avoid asymptotic analysis of the ice response in the far field.

The motion of the cylinder under rigid ice plate was studied. The corresponding equations of motion were integrated analytically using the added mass of a circular cylinder moving near a flat wall. The conformal mapping method was used to solve the hydrodynamic part of the decoupled problem and the Fourier transform method was used to calculate the ice deflection. The leading order speed of deflection and strains in the ice were evaluated thereafter. The decoupled approach is not applicable to the motion of the cylinder in a close proximity of the ice sheet, where the gap between the surface of the cylinder and the deformed ice sheet is comparable with the ice deflection.

It was shown that the presence of a rigid boundary is responsible for a sucking force accelerating the cylinder towards this boundary. At the same time, the added mass, $m_a(h)$, of the cylinder monotonically increases with decrease of the distance *h* from the plate. Light cylinders monotonically approach the plate until impact onto the plate at the speed given by (48). Such light cylinders are accelerated towards the plate by the buoyancy force. Their impact speed monotonically increases with increase of the initial submergence depth. For a heavy cylinder moving vertically towards the plate, there exist a critical Froude number ${{\textrm{Fr}}^*}$ which depends only on the initial dimensionless submergence depth of the cylinder $h_0$. The impact of the cylinder onto the plate occurs for any mass of the cylinder if ${{\textrm{Fr}}}> {{\textrm{Fr}}^*}$ with the impact speed being dependent only on the Froude number ${{\textrm{Fr}}}$ for very heavy cylinders with $M \gg 1$. For small initial speeds of the cylinder moving vertically, ${{\textrm{Fr}}} < {{\textrm{Fr}}^*}$, the impact occurs only if the mass of the cylinder is smaller than a critical value dependent on the angle $\beta$, initial submergence depth $h_0$, and the Froude number ${{\textrm{Fr}}}$.

The present work has been focused on the motions of a cylinder under the ice plate without impacts with the plate. It was shown that the pressure distributed along the plate decreases at the end of the rising stage due to the decrease of the vertical speed of the cylinder. The maximum pressure occurs behind the cylinder during the rising stage and in front of the cylinder during the falling stage. It was found that the maximum pressure is achieved before the cylinder arrives at its highest position, where its vertical speed is zero, see Fig. 8. The strains in the ice plate may exceed the yield strain ${\epsilon _{Y}}$ well before the cylinder starts to move downwards from the plate, see Figs. 9c and 10b. For given radius of the cylinder and its initial kinetic energy, the conditions of the motion including the angle of attack $\beta$ and the dimensionless submergence depth $h_0$ which lead to ice breaking have been predicted, see Fig. 11.

A reader could be surprised how a cylinder with a diameter of 1 meter can break ice 20 cm thick without touching it. It has been confirmed that the ice sheet can significantly bend by the hydrodynamic pressure caused by the movement of underwater objects, see Fig. 1 and results of model tests^5,31–37^. The ice sheet can be broken if the strains in the sheet reach their yield value. The yield strain for the ice is very small, which means that the ice is a rather brittle material. The yield strain in the ice can be achieved even with relatively low hydrodynamic pressure caused by a body moving at a distance from the ice. It is called non-contact ice deformation and breaking induced by a moving load^2,11^. Breaking ice by a moving body has been confirmed in Zemlyak et al. (2013)^33^, see figures from 7 to 11 there, where an underwater object with a cross-section width from 12 to 16 m broke 2 m thick ice.

If the conditions of a cylinder motion are such that the cylinder approaches the ice plate and impacts it at a certain speed, then an impact model should be added to the problem formulation. Impact models can be based either on the Hertz theory or on the Winkler elastic layer approach. Elastic deformations of the plate can be important for the plate/cylinder interaction even before the impact, when the cylinder is moving in a close proximity of the elastic plate.

The presented analysis can be extended to forced motions of circular cylinders under the ice. For higher speeds of the motion, the problem should be solved using the coupled approach. Nonlinear theories of both the ice response and the flow under the ice could be required for special conditions of the ice/body interaction.

## Supplementary Information

Supplementary Information.

## Data Availability

All data generated or analysed during this study are included in this published article.
