# Supplementary material for: Inertial motion of a circular cylinder approaching obliquely an ice cover
Source: Sci Rep. 2025 Mar 19;15:9412. doi: 10.1038/s41598-025-93435-1 (PMC11923159; doi:10.1038/s41598-025-93435-1)
Supplement: Supplementary file 1 — Supplementary Information. [file 41598_2025_93435_MOESM1_ESM.pdf]

Appendix

Conformal mapping approach

The lower half plane, $y < 0$, with a circular hole in it, $z = s - ih + \rho e^{i\alpha}$, where $h > 1, \rho > 1$, is mapped onto a ring, $R < |\zeta| < 1$, in the complex ζ-plane by the mapping,

$$\frac{z-s}{\mu} = i + \frac{2}{\zeta + i}, \tag{79}$$

where $\mu = \sqrt{h^2 - 1}$, $R = h - \mu$ and $\zeta = \xi + i\eta = -ire^{i\theta}$ in the polar coordinates r, θ, see Fig. 12.

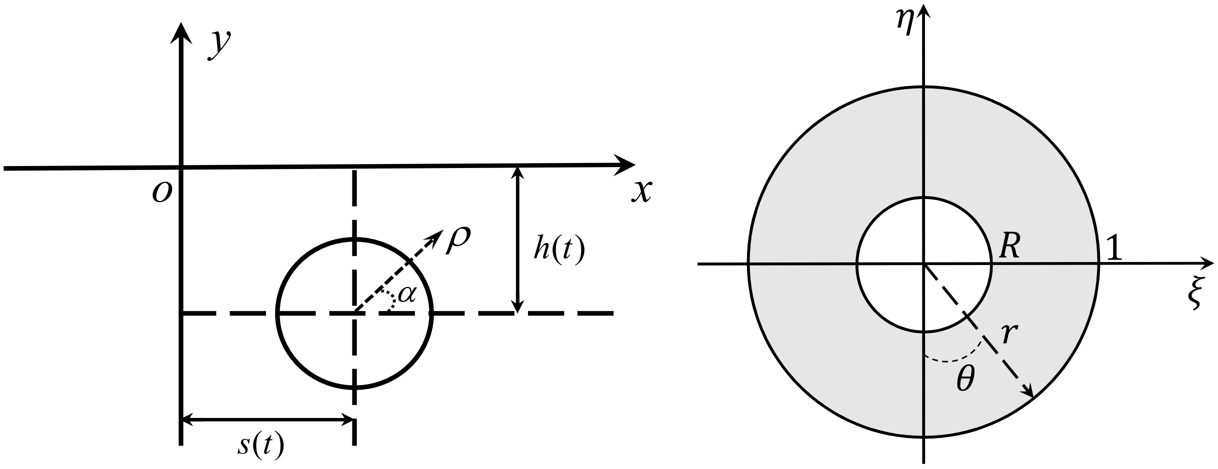

(a) z-plane (b) ζ-plane

Figure 12. The original z-plane and the complex ζ-plane.

Separating the real and imaginary parts in (79), we find the relations between x, y and r, θ,

$$\frac{x - s + iy}{\mu} = \frac{2r\sin\theta + i(r^2 - 1)}{\mathrm{D}}, \tag{80}$$

where $\mathrm{D} = r^2 + 1 - 2r\cos\theta$.
For $r = 1$, which corresponds to the upper boundary, $y = 0$, Eq. (80) provides

$$y = 0, \ \frac{x - s(t)}{\mu(t)} = \frac{\sin\theta}{1 - \cos\theta}. \tag{81}$$

The derivatives $\partial x/\partial r$, $\partial y/\partial r$ at $r = 1$ are calculated by differentiating Eq. (80) in r and setting $r = 1$,

$$\frac{\partial x}{\partial r}(1,\theta,t) = 0, \ \frac{\partial y}{\partial r}(1,\theta,t) = \frac{\mu}{1 - \cos\theta}. \tag{82}$$

Differentiating Eq. (80) in θ at $r = 1$, we find,

$$\frac{\partial y}{\partial \theta}(1,\theta,t) = 0, \ \frac{\partial x}{\partial \theta}(1,\theta,t) = -\frac{\mu}{1 - \cos\theta}. \tag{83}$$

Using the polar coordinates ρ and α, Eq. (80) gives,

$$\frac{\rho\cos\alpha}{\mu} = \frac{2r\sin\theta}{\mathrm{D}}, \quad \frac{-h + \rho\sin\alpha}{\mu} = \frac{r^2 - 1}{\mathrm{D}}. \tag{84}$$

Squaring the sides of (84) and combining them, we obtain,

$$\rho^2\cos^2\alpha + \rho^2\sin^2\alpha = \frac{4\mu^2 r^2\sin^2\theta + (\mu(r^2 - 1) + \mathrm{D}h)^2}{\mathrm{D}^2}, \tag{85}$$

and then

$$\rho(R,\theta,t) = 1, \tag{86}$$

which implies that the circle $r = R$ corresponds to the surface of the cylinder, $\rho = 1$. Eliminating ρ in (84) and setting $r = R$, we find,

$$\cos\alpha = \frac{\mu\sin\theta}{h-\cos\theta}, \sin\alpha = \frac{1-h\cos\theta}{h-\cos\theta}, \tan\alpha = \frac{1-h\cos\theta}{\mu\sin\theta}. \tag{87}$$

Differentiating Eq. (84) in r and setting $r = R$ gives,

$$\frac{\partial\alpha}{\partial r}(R,\theta,t) = 0, \frac{\partial\rho}{\partial r}(R,\theta,t) = \frac{\mu}{R(h-\cos\theta)}. \tag{88}$$

Eq. (86) gives $\rho_\theta(R,\theta,t) = 0$. Differentiating the first formula of Eq. (84) in θ and setting $r = R$, we find

$$\alpha_\theta(R,\theta,t) = \frac{\mu}{h-\cos\theta}. \tag{89}$$

The potential function $\phi(r,\theta,t)$ defined by Eq. (23) satisfies Laplace's equation in the ring $R < r < 1$. On the circle $r = 1$, we have

$$\frac{\partial\phi}{\partial r}(1,\theta,t) = \frac{\partial\varphi}{\partial x}\frac{\partial x}{\partial r}(1,\theta,t) + \frac{\partial\varphi}{\partial y}\frac{\partial y}{\partial r}(1,\theta,t) = 0, \tag{90}$$

where $\partial x/\partial r(1,\theta,t) = 0$, see (82), and $\partial\varphi/\partial y(x,0,t) = 0$, see the boundary condition (19). On the inner circle $r = R$, Eqs. (88) give

$$\frac{\partial\phi}{\partial r}(R,\theta,t) = \frac{\partial\varphi}{\partial\rho}\frac{\partial\rho}{\partial r}(R,\theta,t) + \frac{\partial\varphi}{\partial\alpha}\frac{\partial\alpha}{\partial r}(R,\theta,t) = \frac{\partial\varphi}{\partial\rho}[1,\alpha(R,\theta,t),t]\frac{\mu}{R(h-\cos\theta)} = \dot{s}\frac{\mu^2}{R}\hat{f}_1(\theta,t) - \dot{h}\frac{\mu}{R}\hat{f}_2(\theta,t), \tag{91}$$

where

$$\hat{f}_1(\theta,t) = \frac{\sin\theta}{(h-\cos\theta)^2}, \quad \hat{f}_2(\theta,t) = \frac{1-h\cos\theta}{(h-\cos\theta)^2}, \tag{92}$$

$\hat{f}_1(\theta,t)$ and $\hat{f}_2(\theta,t)$ are odd and even functions of θ respectively. They can be presented by their Fourier series,

$$\hat{f}_1(\theta,t) = \sum_{n=1}^{\infty} f_{1n}(h)\sin(n\theta), \quad \hat{f}_2(\theta,t) = \frac{f_{20}(h)}{2} + \sum_{n=1}^{\infty} f_{2n}(h)\cos(n\theta), \tag{93}$$

where

$$f_{1n}(h) = \frac{1}{\pi}\int_{-\pi}^{\pi}\hat{f}_1(\theta)\sin n\theta \mathrm{d}\theta = \frac{n}{\pi}I_n(h), \tag{94}$$

$$f_{2n}(h) = \frac{1}{\pi}\int_{-\pi}^{\pi}\hat{f}_2(\theta)\cos n\theta \mathrm{d}\theta = \frac{1}{\pi}J_n(h). \tag{95}$$

Here

$$I_n(h) = \int_{-\pi}^{\pi}\frac{\cos n\theta}{h-\cos\theta}\mathrm{d}\theta, \quad J_n(h) = \int_{-\pi}^{\pi}\frac{(1-h\cos\theta)\cos n\theta}{(h-\cos\theta)^2}\mathrm{d}\theta.$$

The functions $I_n(h)$ and $J_n(h)$ can be written as contour integrals by using $z = e^{i\theta}$, $\mathrm{d}\theta = \mathrm{d}z/(iz)$ and $\cos\theta = (z+1/z)/2$,

$$I_n(h) = -\frac{2}{i}\oint_{|z|=1}\frac{z^n}{z^2-2hz+1}\mathrm{d}z = -\frac{2}{i}\oint_{|z|=1}\frac{z^n}{(z-R)(z-1/R)}\mathrm{d}z,$$

where $z^2 - 2zh + 1 = (z-h-\mu)(z+\mu-h) = (z-1/R)(z-R)$. The function under the integral has the first-order pole at $z = R$ inside the unit circle $|z| = 1$, then by the residue theorem,

$$I_n(h) = -\frac{2}{i}\left\{2\pi i\,\mathrm{Res}\left[\frac{z^n}{(z-R)(z-1/R)}, R\right]\right\} = \frac{2\pi R^n}{\mu}. \tag{96}$$

where it was used that $1 - R^2 = 2\mu R$. Correspondingly,

$$J_n(h) = \frac{2}{i}\oint_{|z|=1}\frac{2z-h(z^2+1)}{(z-R)^2(z-1/R)^2}z^n\mathrm{d}z,$$

where $z = R$ inside the unit circle $|z| = 1$ is the second-order pole. The residue theorem provides

$$J_n(h) = \frac{2}{i}\left\{2\pi i \operatorname{Res}\left[\frac{2z - h(z^2+1)}{(z-R)^2(z-1/R)^2}, R\right]\right\} = -2n\pi R^n, \tag{97}$$

where

$$\operatorname{Res}\left[\frac{2z - h(z^2+1)}{(z-R)^2(z-1/R)^2}, R\right] = \lim_{z\to R}\frac{\mathrm{d}}{\mathrm{d}z}\left(\frac{2z-h(z^2+1)}{(z-1/R)^2}z^n\right) = -\frac{n}{2}R^n.$$

Substituting (96), (97) into (94), (95) and then (91) gives,

$$\frac{\partial\phi}{\partial r}(R,\theta,t) = \dot{s}f_1(\theta,t) - \dot{h}f_2(\theta,t), \tag{98}$$

where

$$f_1(\theta,t) = \frac{2\mu}{R}\sum_{n=1}^{\infty} nR^n\sin(n\theta),\ f_2(\theta,t) = -\frac{2\mu}{R}\sum_{n=1}^{\infty} nR^n\cos(n\theta). \tag{99}$$

Dimensionless added mass

The integrals in (37) and (38) with respect to θ are denoted as $c_1(h)$ and $c_2(h)$,

$$c_1(h) = \mu^2\int_{-\pi}^{\pi}\phi_1(R,\theta,h)\frac{\sin\theta}{(h-\cos\theta)^2}\mathrm{d}\theta,\ c_2(h) = \mu\int_{-\pi}^{\pi}\phi_2(R,\theta,h)\frac{h\cos\theta - 1}{(h-\cos\theta)^2}\mathrm{d}\theta. \tag{100}$$

Eqs. (27) and (28) at $r = R$ yield

$$\phi_1(R,\theta,h) = -2\mu\sum_{n=1}^{\infty}\frac{1+R^{2n}}{R^n}\varphi_n\sin(n\theta),\ \phi_2(R,\theta,h) = 2\mu\sum_{n=1}^{\infty}\varphi_n(\frac{1+R^{2n}}{R^n}\cos(n\theta) - 2), \tag{101}$$

where $\varphi_n(h) = R^{2n}/(1-R^{2n})$. Substituting (101) in (100), we obtain,

$$c_1(h) = -2\mu^3\sum_{n=1}^{\infty}\frac{1+R^{2n}}{R^n}\varphi_n\int_{-\pi}^{\pi}\frac{\sin(n\theta)\sin\theta}{(h-\cos\theta)^2}\mathrm{d}\theta = -\pi m_a(h), \tag{102}$$

and

$$c_2(h) = -2\mu^2\sum_{n=1}^{\infty}\frac{1+R^{2n}}{R^n}\varphi_n J_n(h) + 4\mu^2\sum_{n=1}^{\infty}\varphi_n J_0(h) = \pi m_a(h), \tag{103}$$

where $J_n(h)$, $n \geq 0$, are given by (97),

$$m_a(h) = 4\mu^2\sum_{n=1}^{\infty} nR^{2n}\frac{1+R^{2n}}{1-R^{2n}} \tag{104}$$

and

$$\int_{-\pi}^{\pi}\frac{\sin(n\theta)\sin\theta}{(h-\cos\theta)^2}\mathrm{d}\theta = \frac{2\pi nR^n}{\mu},$$

which is calculated in the same way as the integrals $I_n(h)$ and $J_n(h)$ in (96) and (97).

The integration in Eq.(41) gives

$$\int_{-\infty}^{\infty}\varphi_x^2(x,0,t)\mathrm{d}x = -\int_{-\pi}^{\pi}\phi_\theta^2(1,\theta,t)\frac{\mathrm{d}\theta}{x_\theta}, \tag{105}$$

where $x \to -\infty$ corresponds to $\theta \to -0$, $x \to -0$ corresponds to $\theta = -\pi$, $x \to +0$ corresponds to $\theta = \pi$ and $x \to \infty$ corresponds to $\theta = +0$. Differentiating (24) in θ, $\phi_\theta(r,\theta,t) = \dot{s}(t)\phi_{1\theta}(r,\theta,h) - \dot{h}(t)\phi_{2\theta}(r,\theta,h)$, and using (27), (28), we find that

$$\phi_{1\theta}(1,\theta,h) = -4\mu\sum_{n=1}^{\infty} n\varphi_n(h)\cos(n\theta)$$

is an even function of θ and

$$\phi_{2\theta}(1,\theta,h) = -4\mu \sum_{n=1}^{\infty} n\varphi_n(h)\sin(n\theta)$$

is an odd function of θ. Noting that $x_\theta(1,\theta,t)$ is an even function of θ, see (83), we obtain,

$$\int_{-\infty}^{\infty} \varphi_x^2(x,0,t)\mathrm{d}x = 16\mu\left(\dot{s}^2+\dot{h}^2\right)\sum_{n=1,\,m=1}^{\infty} nm\varphi_n\varphi_m L_{nm}, \tag{106}$$

where

$$L_{nm} = \int_{-\pi}^{\pi}\cos(n\theta)\cos(m\theta)(1-\cos\theta)\,\mathrm{d}\theta = \int_{-\pi}^{\pi}\sin(n\theta)\sin(m\theta)(1-\cos\theta)\,\mathrm{d}\theta$$

and then

$$L_{nm} = \pi\delta_{n,m} - \frac{1}{2}\pi(\delta_{n,m+1}+\delta_{m,n+1}), \tag{107}$$

where $\delta_{n,m}=1$ for $n=m$, $\delta_{n,m}=0$ for $n\neq m$.
Substituting (107) in (106) gives

$$\int_{-\infty}^{\infty} \varphi_x^2(x,0,t)\mathrm{d}x = 2\pi(\dot{s}^2+\dot{h}^2)K(h),\; K(h) = 8\mu\sum_{n=1}^{\infty} n\varphi_n(n\varphi_n-(n+1)\varphi_{n+1}). \tag{108}$$

It will be shown below that $K(h) = -\frac{1}{2}\mathrm{d}m_a/\mathrm{d}h$, where $m_a(h)$ is given by (104), $\mu=\sqrt{h^2-1}$ and $R=h-\mu$. We start with the derivative of (104),

$$\frac{\mathrm{d}m_a(h)}{\mathrm{d}h} = 8h\sum_{n=1}^{\infty} n\varphi_n\left(1+R^{2n}\right) - 8\mu\sum_{n=1}^{\infty} n^2\left[\varphi_n + 2R^{2n}\varphi_n + \varphi_n^2\left(1+R^{2n}\right)\right], \tag{109}$$

where we used that $\mathrm{d}\mu/\mathrm{d}h = h/\mu$, $\mathrm{d}R/\mathrm{d}h = -R/\mu$. Using $1+R^{2n} = -\left(1-R^{2n}\right)+2$ and $R^{2n} = -\left(1-R^{2n}\right)+1$ in (109), we find

$$\frac{\mathrm{d}m_a(h)}{\mathrm{d}h} = 16h\sum_{n=1}^{\infty} n\varphi_n - 16\mu\sum_{n=1}^{\infty} n^2\varphi_n - 16\mu\sum_{n=1}^{\infty} n^2\varphi_n^2 + 8\mu\sum_{n=1}^{\infty} n^2R^{2n} - 8h\sum_{n=1}^{\infty} nR^{2n}, \tag{110}$$

where

$$\begin{aligned} 8\mu\sum_{n=1}^{\infty} n^2R^{2n} - 8h\sum_{n=1}^{\infty} nR^{2n} &= 8\mu\frac{R^2\left(1+R^2\right)}{(1-R^2)^3} - 8h\frac{R^2}{(1-R^2)^2} = \frac{8R^2}{(1-R^2)^3}\left[\mu\left(1+R^2\right)-h\left(1-R^2\right)\right] \\ &= \frac{8R^2}{(1-R^2)^3}\left[-R+R^2(\mu+h)\right] = \frac{8R^3}{(1-R^2)^3}\left[(h-\mu)(h+\mu)-1\right] = 0. \end{aligned} \tag{111}$$

To prove (111) we used the following formulas, $\sum_{n=0}^{\infty} x^n = \frac{1}{1-x}$, $\sum_{n=1}^{\infty} nx^n = \frac{x}{(1-x)^2}$, $\sum_{n=1}^{\infty} n^2x^n = \frac{x(1+x)}{(1-x)^3}$, where $x=R^2$, and equality $h^2-\mu^2-1=0$.

The result is presented in the form

$$-\frac{1}{2}\frac{\mathrm{d}m_a}{\mathrm{d}h} = 8\mu\sum_{n=1}^{\infty} n^2\varphi_n^2 + 8\mu\sum_{n=1}^{\infty} n^2\varphi_n - 8h\sum_{n=1}^{\infty} n\varphi_n. \tag{112}$$

The first term on the right-hand side of (112) is equal to the first term in the formula (108) for $K(h)$. The second series in (108) gives

$$\varphi_n\varphi_{n+1} = \frac{R^{2n+2}}{1-R^2}\left(\frac{1}{1-R^{2n}} - \frac{1}{1-R^{2n+2}}\right)$$

and

$$L = \sum_{n=1}^{\infty} n(n+1)\varphi_n\varphi_{n+1} = \frac{R^2}{1-R^2}\sum_{n=1}^{\infty} n(n+1)\varphi_n - \frac{R^2}{1-R^2}\sum_{n=1}^{\infty} n(n+1)\frac{R^{2n}}{1-R^{2n+2}}. \tag{113}$$

Introducing $n+1=m$ in the second series of (113), we find

$$L = \frac{R^2}{1-R^2}\sum_{n=1}^{\infty} n\,(n+1)\,\varphi_n - \frac{R^2}{1-R^2}\sum_{m=2}^{\infty} m\,(m-1)\,\frac{R^{2m-2}}{1-R^{2m}}.$$

In the second series, we can start summation from $m=1$ because the corresponding term with $m=1$ is equal to zero. Then we change m to n in the series,

$$\begin{aligned} L &= \frac{R^2}{1-R^2}\sum_{n=1}^{\infty} n\,(n+1)\,\varphi_n - \frac{1}{1-R^2}\sum_{n=1}^{\infty} n\,(n-1)\,\varphi_n \\ &= -\sum_{n=1}^{\infty} n^2\varphi_n + \frac{1+R^2}{1-R^2}\sum_{n=1}^{\infty} n\varphi_n = -\sum_{n=1}^{\infty} n^2\varphi_n + \frac{h}{\mu}\sum_{n=1}^{\infty} n\varphi_n \end{aligned} \tag{114}$$

where $\dfrac{1+R^2}{1-R^2} = \dfrac{1+(h-\mu)^2}{1-(h-\mu)^2} = \dfrac{2h^2-2\mu h}{-2\mu^2+2\mu h} = \dfrac{h}{\mu}$.

Substituting (114) in the formula (108) for $K(h)$, we obtain

$$K(h) = 8\mu\sum_{n=1}^{\infty} n^2\varphi_n^2 + 8\mu\sum_{n=1}^{\infty} n^2\varphi_n - 8h\sum_{n=1}^{\infty} n\varphi_n,$$

which is the same as the right-hand side of (112). Therefore,

$$K(h) = -\frac{1}{2}\frac{\mathrm{d}m_a}{\mathrm{d}h}. \tag{115}$$
